# Supplementary material for: Fidelity in co-diversified symbiosis
Source: Nat Commun. 2026 Feb 12;17:1644. doi: 10.1038/s41467-026-69366-4 (PMC12905250; doi:10.1038/s41467-026-69366-4)
Supplement: Supplementary file 1 — Supplementary Information [file 41467_2026_69366_MOESM1_ESM.pdf]

Supplementary Information

Fidelity in co-diversified symbiosis

Inès Pons [1,*], Marleny García-Lozano [1], Christiane Emmerich [1], Aftab Mahmood Ayas [1], Christine Henzler [1], Hagay Enav [2], Ruth Ley [2], Hassan Salem [1,*]

[1] Mutualisms Research Group, Max Planck Institute for Biology, Tübingen 72076, Germany

[2] Department of Microbiome Science, Max Planck Institute for Biology, Tübingen 72076, Germany

* For correspondence: ines.pons-guillouard@tuebingen.mpg.de; hassan.salem@tuebingen.mpg.de

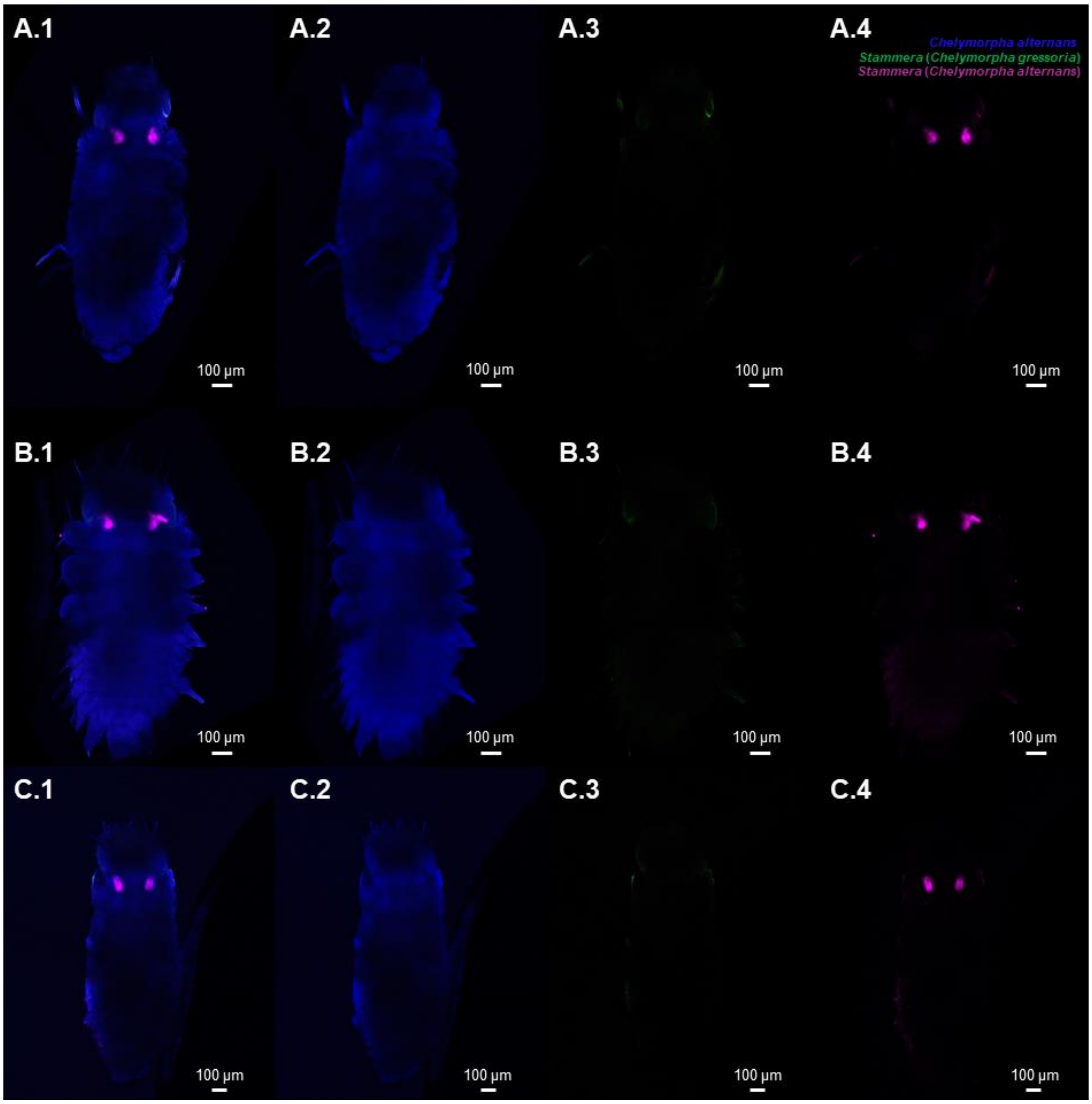

Figure S1i (A-C)**.** Fluorescence *in situ* hybridization (FISH) replicates on whole-mounts of *Chelymorpha alternans* embryos (untreated control) (*n* = 3 rep). Probes used: *Chelymorpha alternans* host (blue: 18S rRNA), *Stammera* from *Chelymorpha gressoria* (green: 16SrRNA), and *Stammera* from *Chelymorpha alternans* (magenta: 16S rRNA). **(1)** correspond to the merged channels images while the others correspond to individual channel images: **(2)** host probe, **(3)** *Stammera* from *Chelymorpha gressoria* probe, and **(4)** *Stammera* from *Chelymorpha alternans* probe. Scale bars (100 µm) are included for reference.

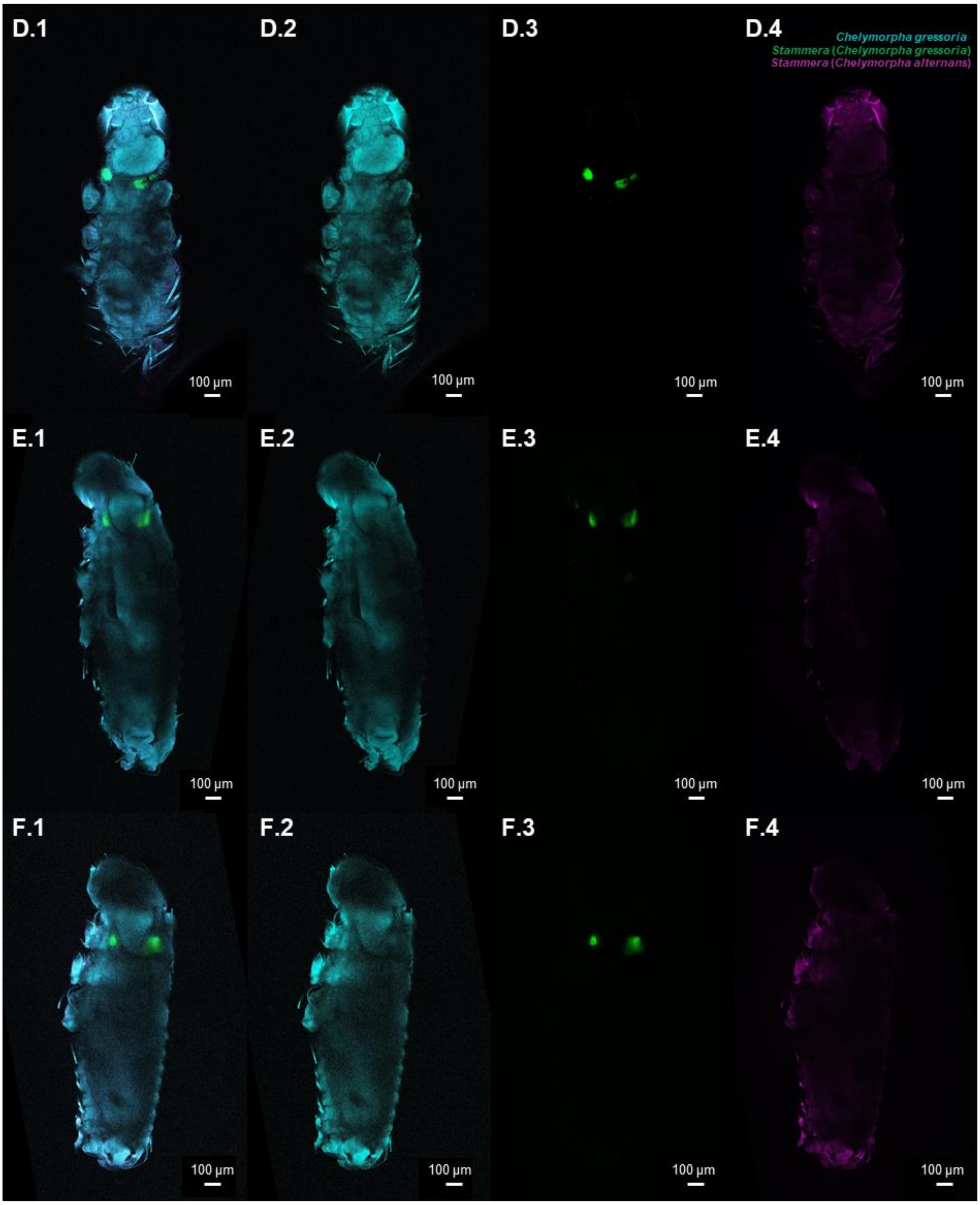

Figure S1i (D-F)**.** Fluorescence *in situ* hybridization (FISH) replicates on whole-mounts of *Chelymorpha gressoria* embryos (untreated control) (*n* = 3 rep). Probes used: *Chelymorpha gressoria* host (cyan: 18S rRNA), *Stammera* from *Chelymorpha gressoria* (green: 16SrRNA), and *Stammera* from *Chelymorpha alternans* (magenta: 16S rRNA). **(1)** correspond to the merged channels images while the others correspond to individual channel images: **(2)** host probe, **(3)** *Stammera* from *Chelymorpha gressoria* probe, and **(4)** *Stammera* from *Chelymorpha alternans* probe. Scale bars (100 µm) are included for reference.

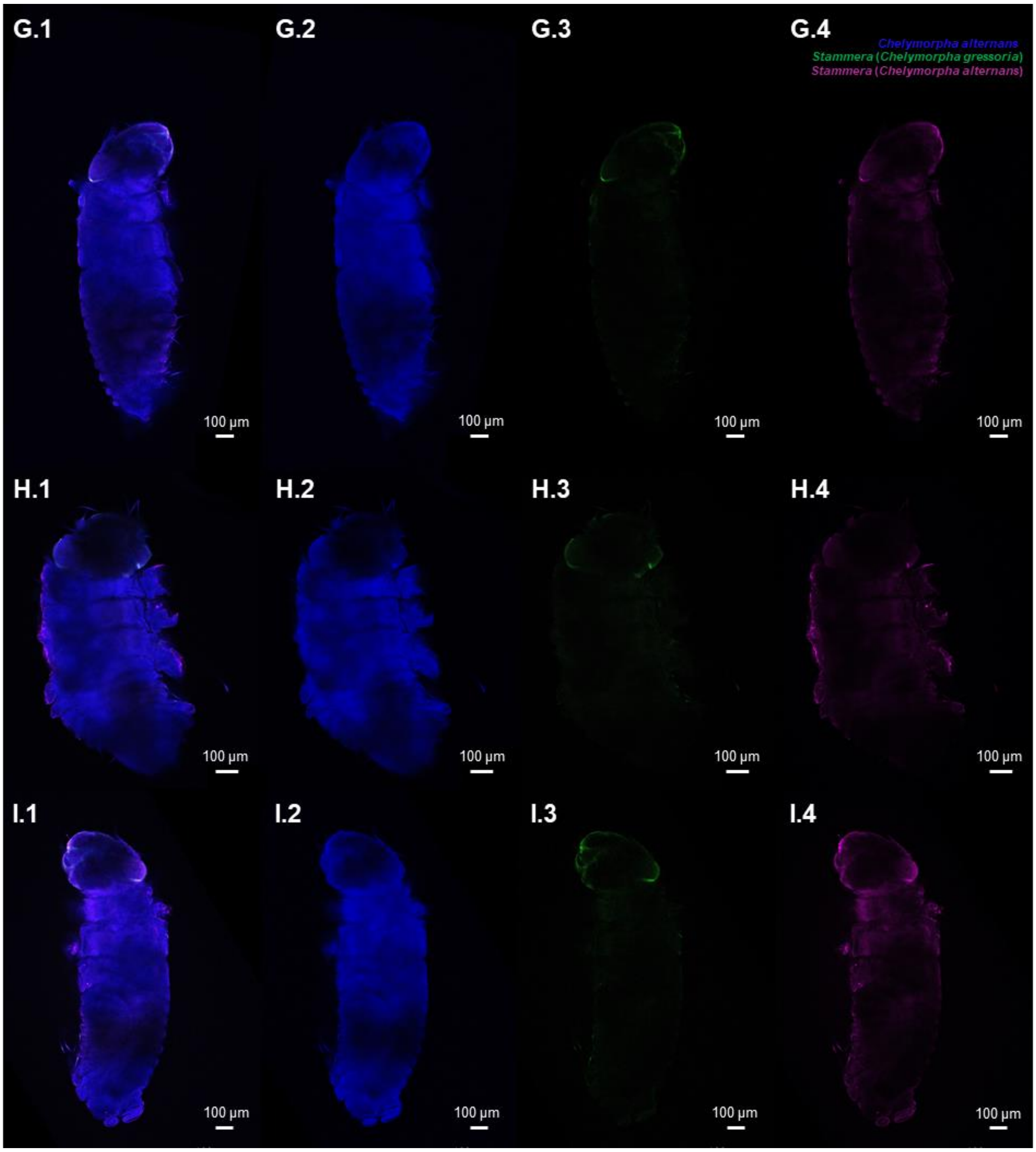

Figure S1i (G-I)**.** Fluorescence *in situ* hybridization (FISH) replicates on whole-mounts of *Chelymorpha alternans* embryos (aposymbiotic) (*n* = 3 rep). Probes used: *Chelymorpha alternans* host (blue: 18S rRNA), *Stammera* from *Chelymorpha gressoria* (green: 16SrRNA), and *Stammera* from *Chelymorpha alternans* (magenta: 16S rRNA). **(1)** correspond to the merged channels images while the others correspond to individual channel images: **(2)** host probe, **(3)** *Stammera* from *Chelymorpha gressoria* probe, and **(4)** *Stammera* from *Chelymorpha alternans* probe. Scale bars (100 μm) are included for reference.

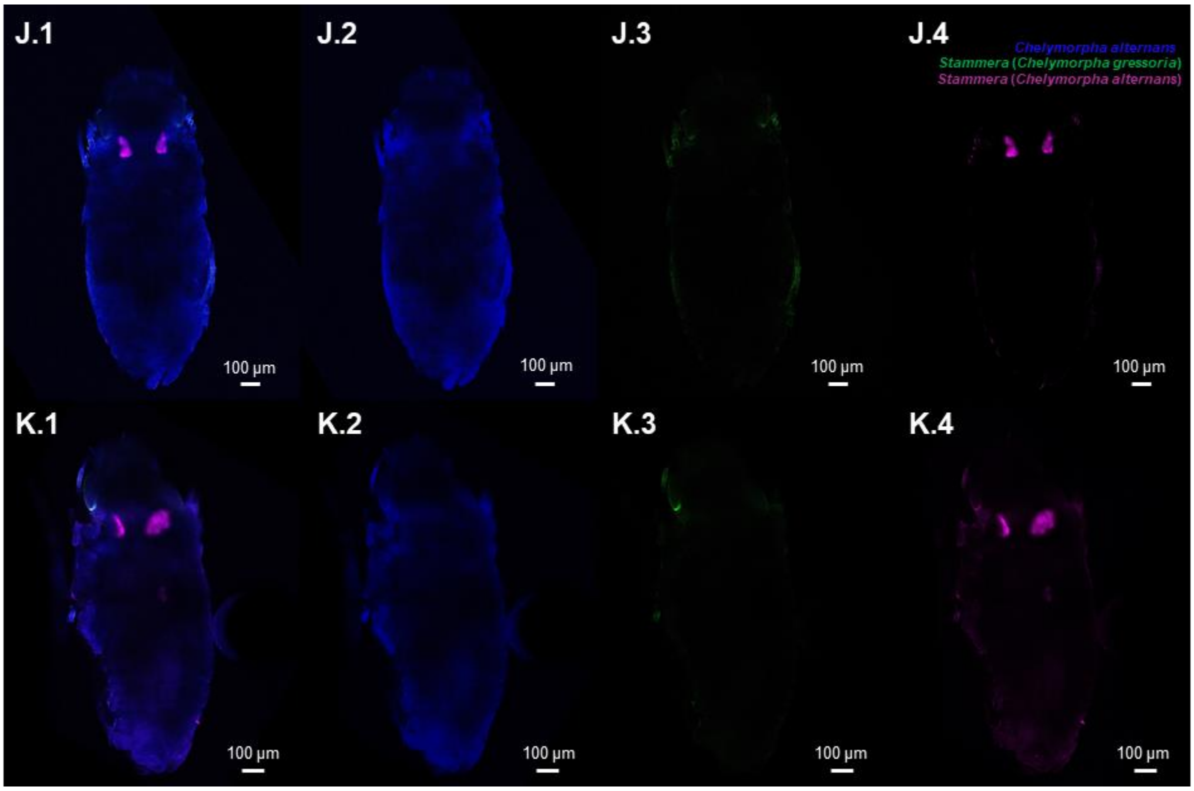

Figure S1i (J-K)**.** Fluorescence *in situ* hybridization (FISH) replicates on whole-mounts of *Chelymorpha alternans* embryos (re-infected) (*n* = 3 rep). Probes used: *Chelymorpha alternans* host (blue: 18S rRNA), *Stammera* from *Chelymorpha gressoria* (green: 16SrRNA), and *Stammera* from *Chelymorpha alternans* (magenta: 16S rRNA). **(1)** correspond to the merged channels images while the others correspond to individual channel images: **(2)** host probe, **(3)** *Stammera* from *Chelymorpha gressoria* probe, and **(4)** *Stammera* from *Chelymorpha alternans* probe. Scale bars (100 µm) are included for reference.

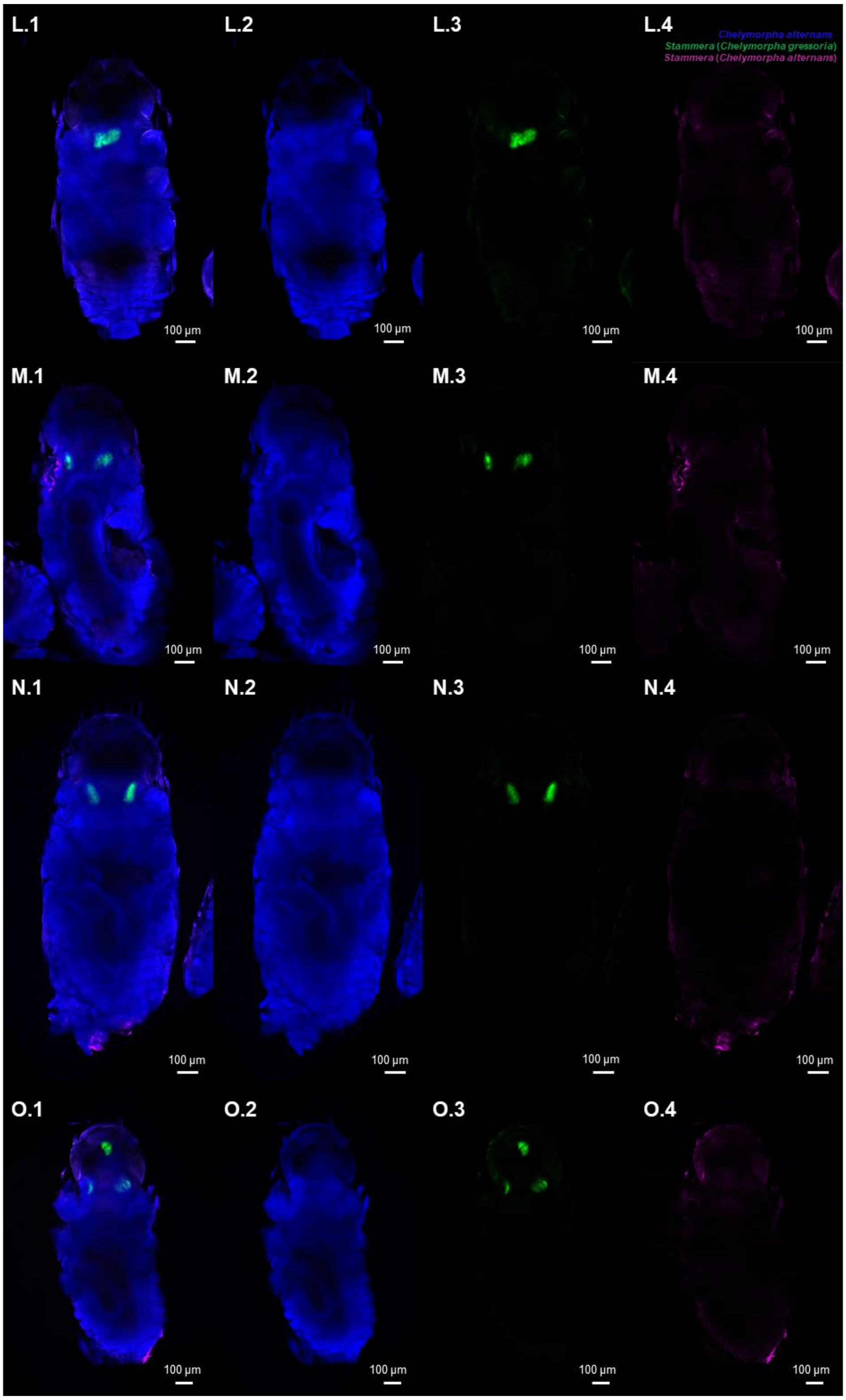

Figure S1i (L-O)**.** Fluorescence *in situ* hybridization (FISH) replicates on whole-mounts of *Chelymorpha alternans* embryos (cross-infected) (*n* = 4 rep). Probes used: *Chelymorpha alternans* host (blue: 18S rRNA), *Stammera* from *Chelymorpha gressoria* (green: 16SrRNA), and *Stammera* from *Chelymorpha alternans* (magenta: 16S rRNA). **(1)** correspond to the merged channels images while the others correspond to individual channel images: **(2)** host probe, **(3)** *Stammera* from *Chelymorpha gressoria* probe, and **(4)** *Stammera* from *Chelymorpha alternans* probe. Scale bars (100 µm) are included for reference.

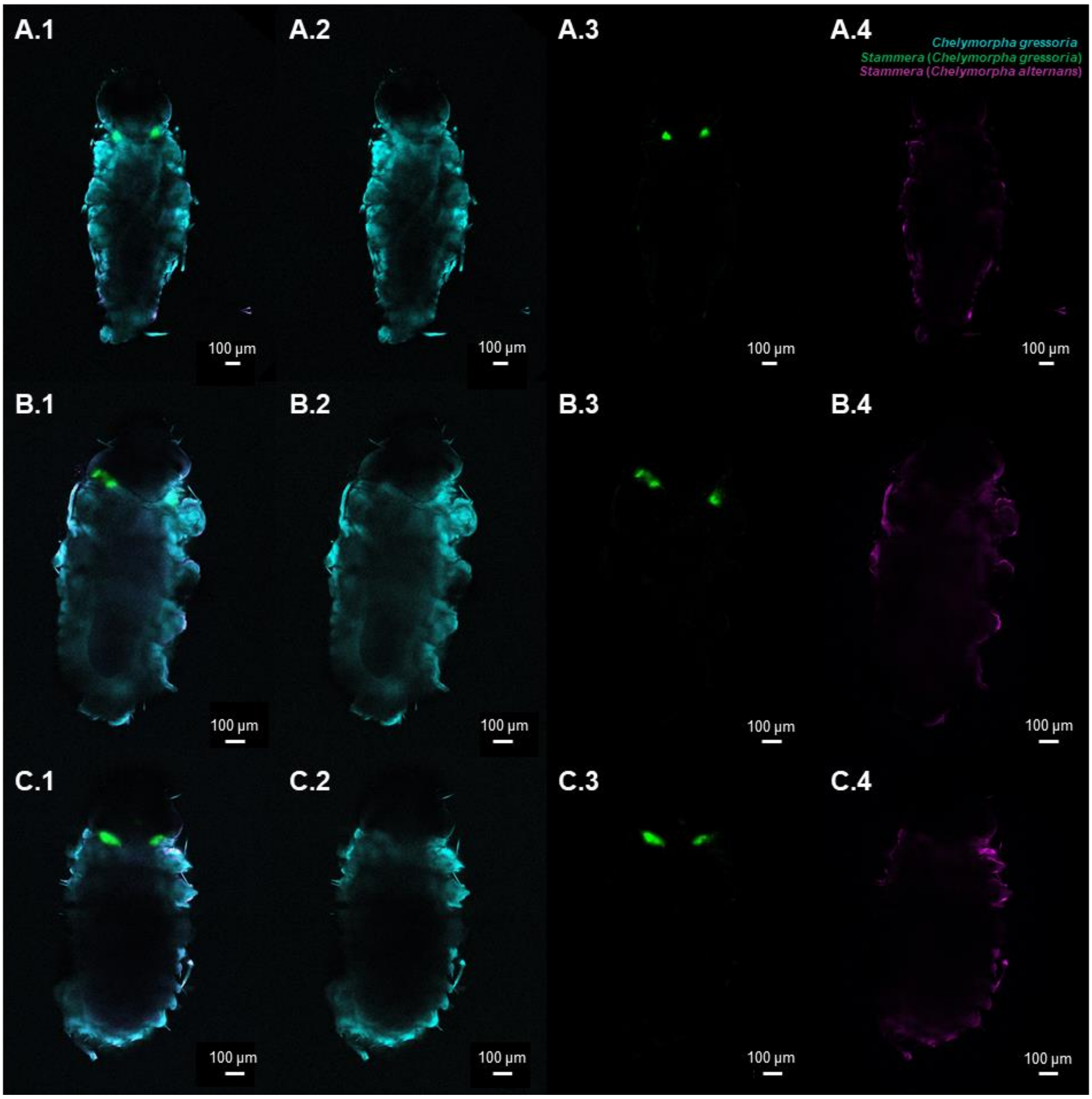

Figure S1ii (A-C)**.** Fluorescence *in situ* hybridization (FISH) replicates on whole-mounts of *Chelymorpha gressoria* embryos (untreated control) (*n* = 3 rep). Probes used: *Chelymorpha gressoria* host (cyan: 18S rRNA), *Stammera* from *Chelymorpha gressoria* (green: 16SrRNA), and *Stammera* from *Chelymorpha alternans* (magenta: 16S rRNA). **(1)** correspond to the merged channels images while the others correspond to individual channel images: **(2)** host probe, **(3)** *Stammera* from *Chelymorpha gressoria* probe, and **(4)** *Stammera* from *Chelymorpha alternans* probe. Scale bars (100 µm) are included for reference.

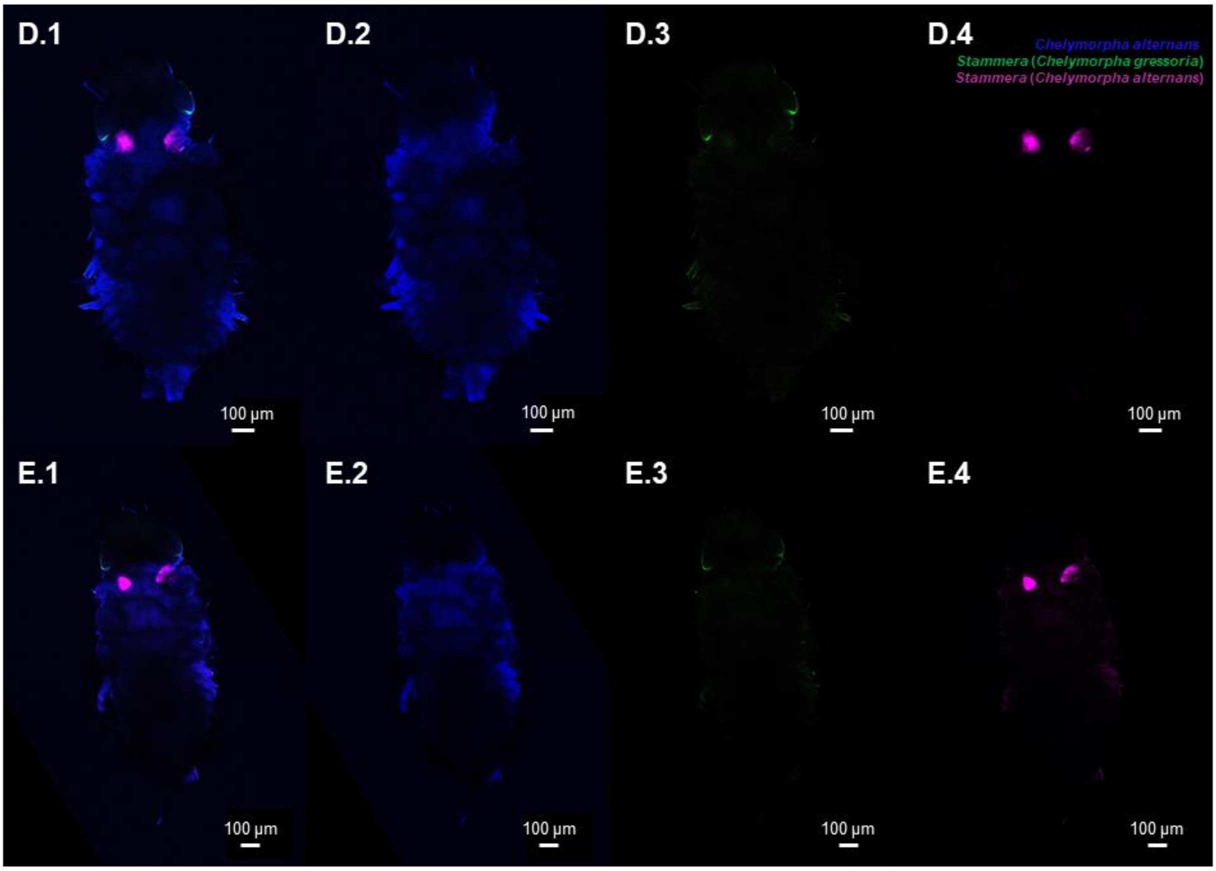

Figure S1ii (D-E)**.** Fluorescence *in situ* hybridization (FISH) replicates on whole-mounts of *Chelymorpha alternans* embryos (untreated control) (*n* = 2 rep). Probes used: *Chelymorpha alternans* host (blue: 18S rRNA), *Stammera* from *Chelymorpha gressoria* (green: 16SrRNA), and *Stammera* from *Chelymorpha alternans* (magenta: 16S rRNA). **(1)** correspond to the merged channels images while the others correspond to individual channel images: **(2)** host probe, **(3)** *Stammera* from *Chelymorpha gressoria* probe, and **(4)** *Stammera* from *Chelymorpha alternans* probe. Scale bars (100 μm) are included for reference.

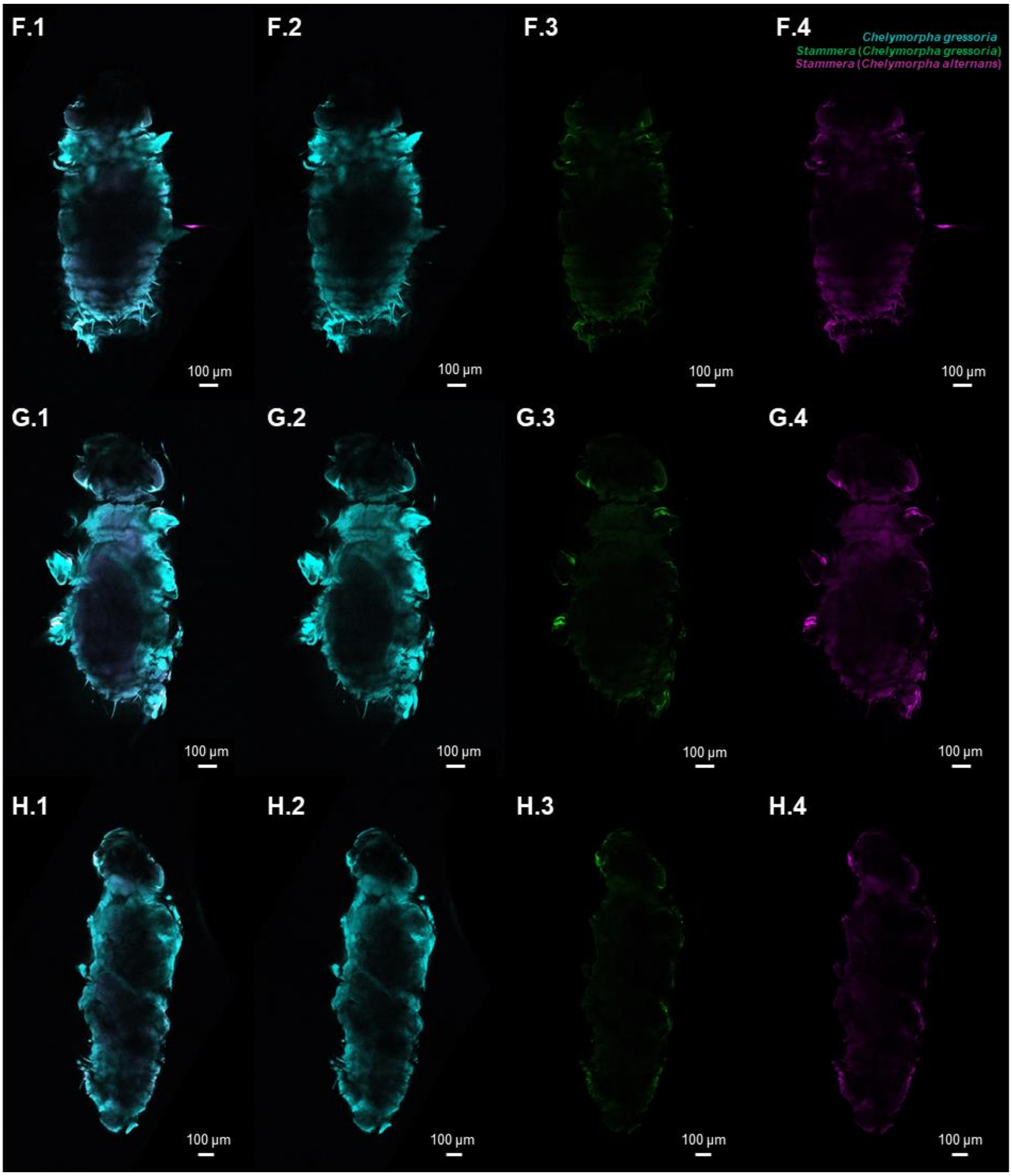

Figure S1ii (F-H)**.** Fluorescence *in situ* hybridization (FISH) replicates on whole-mounts of *Chelymorpha gressoria* embryos (aposymbiotic) (*n* = 3 rep). Probes used: *Chelymorpha gressoria* host (cyan: 18S rRNA), *Stammera* from *Chelymorpha gressoria* (green: 16SrRNA), and *Stammera* from *Chelymorpha alternans* (magenta: 16S rRNA). **(1)** correspond to the merged channels images while the others correspond to individual channel images: **(2)** host probe, **(3)** *Stammera* from *Chelymorpha gressoria* probe, and **(4)** *Stammera* from *Chelymorpha alternans* probe. Scale bars (100 µm) are included for reference.

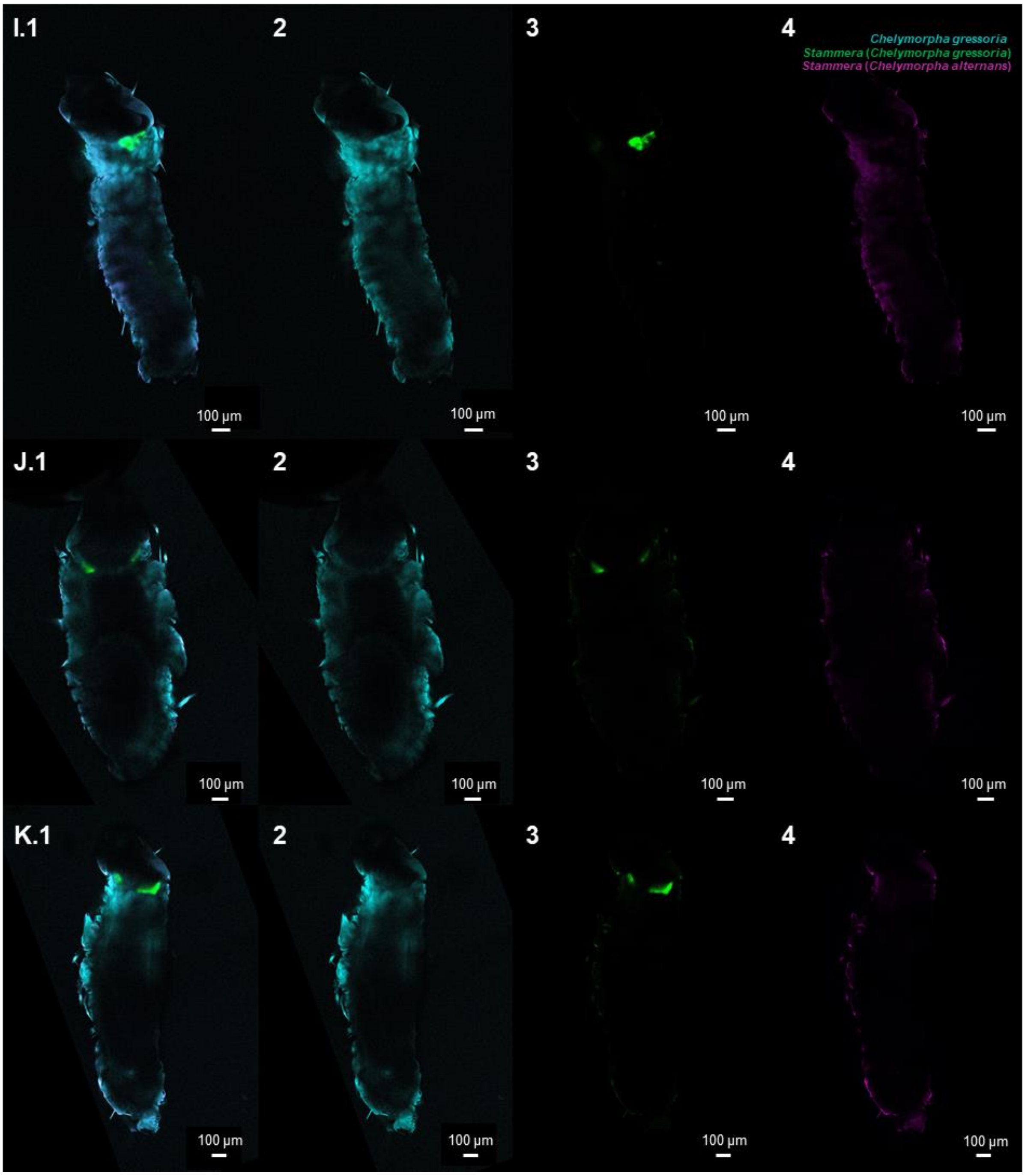

Figure S1ii (I-K)**.** Fluorescence *in situ* hybridization (FISH) replicates on whole-mounts of *Chelymorpha gressoria* embryos (re-infected) (*n* = 3 rep). Probes used: *Chelymorpha gressoria* host (cyan: 18S rRNA), *Stammera* from *Chelymorpha gressoria* (green: 16SrRNA), and *Stammera* from *Chelymorpha alternans* (magenta: 16S rRNA). **(1)** correspond to the merged channels images while the others correspond to individual channel images: **(2)** host probe, **(3)** *Stammera* from *Chelymorpha gressoria* probe, and **(4)** *Stammera* from *Chelymorpha alternans* probe. Scale bars (100 µm) are included for reference.

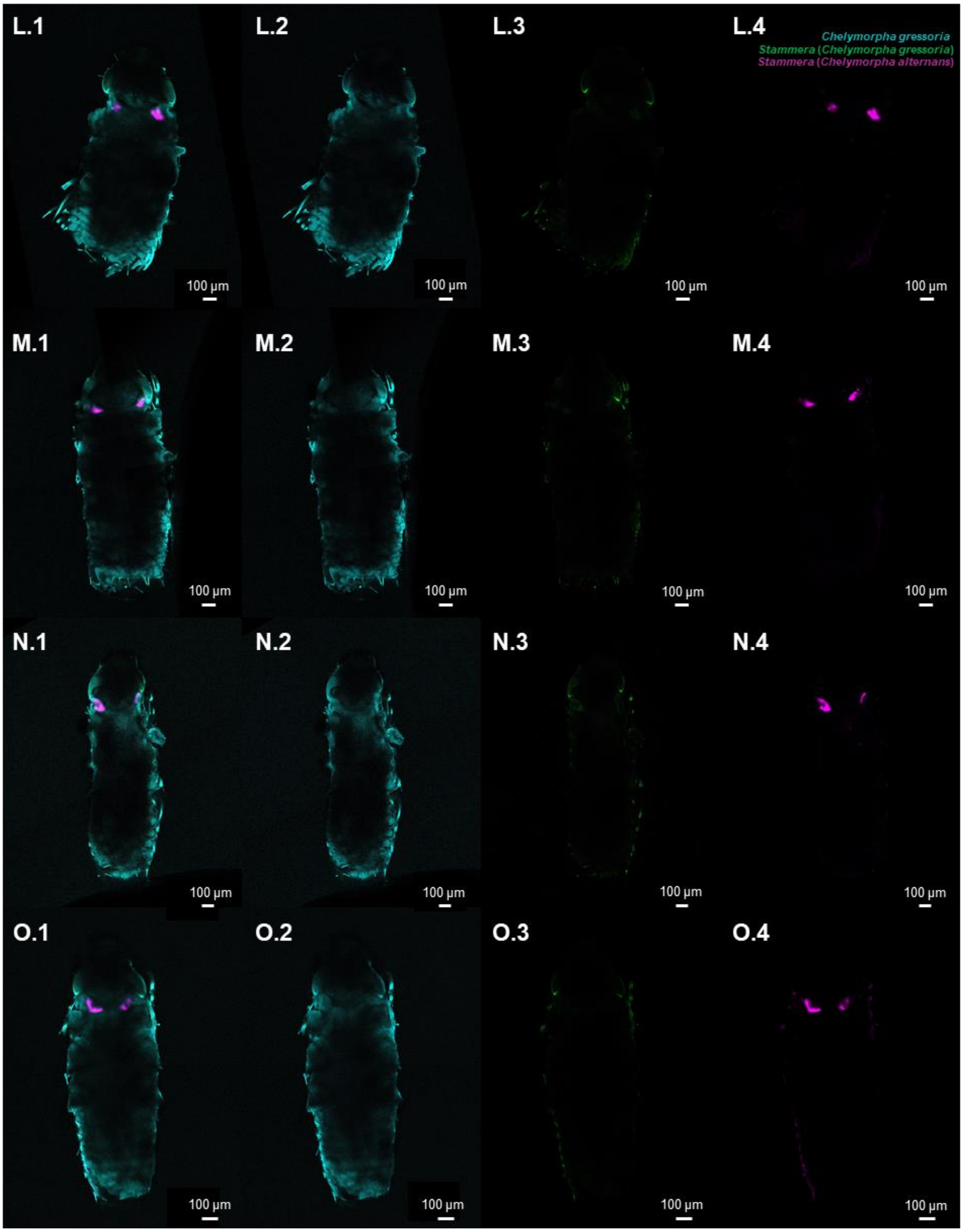

Figure S1ii (L-O)**.** Fluorescence *in situ* hybridization (FISH) replicates on whole-mounts of *Chelymorpha gressoria* embryos (cross-infected) (*n* = 4 rep). Probes used: *Chelymorpha gressoria* host (cyan: 18S rRNA), *Stammera* from *Chelymorpha gressoria* (green: 16SrRNA), and *Stammera* from *Chelymorpha alternans* (magenta: 16S rRNA). **(1)** correspond to the merged channels images while the others correspond to individual channel images: **(2)** host probe, **(3)** *Stammera* from *Chelymorpha gressoria* probe, and **(4)** *Stammera* from *Chelymorpha alternans* probe. Scale bars (100 µm) are included for reference.

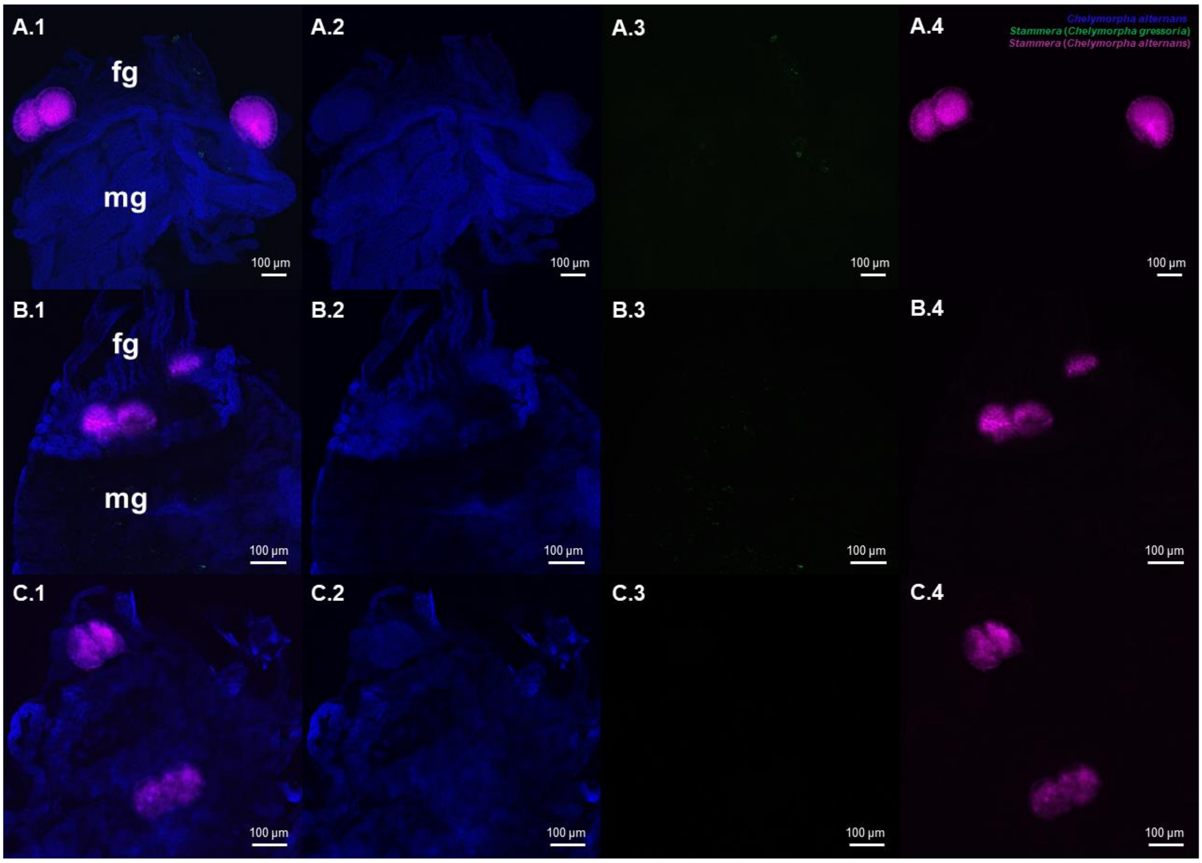

Figure S1iii (A-C)**.** Fluorescence *in situ* hybridization (FISH) replicates on whole-mounts of *Chelymorpha alternans* larvae foregut symbiotic organs (untreated control) (*n* = 3 rep). Probes used: *Chelymorpha alternans* host (blue: 18S rRNA), *Stammera* from *Chelymorpha gressoria* (green: 16SrRNA), and *Stammera* from *Chelymorpha alternans* (magenta: 16S rRNA). **(1)** correspond to the merged channels images while the others correspond to individual channel images: **(2)** host probe, **(3)** *Stammera* from *Chelymorpha gressoria* probe, and **(4)** *Stammera* from *Chelymorpha alternans* probe. Abbreviations: fg, foregut; mg, midgut. Scale bars (100 µm) are included for reference.

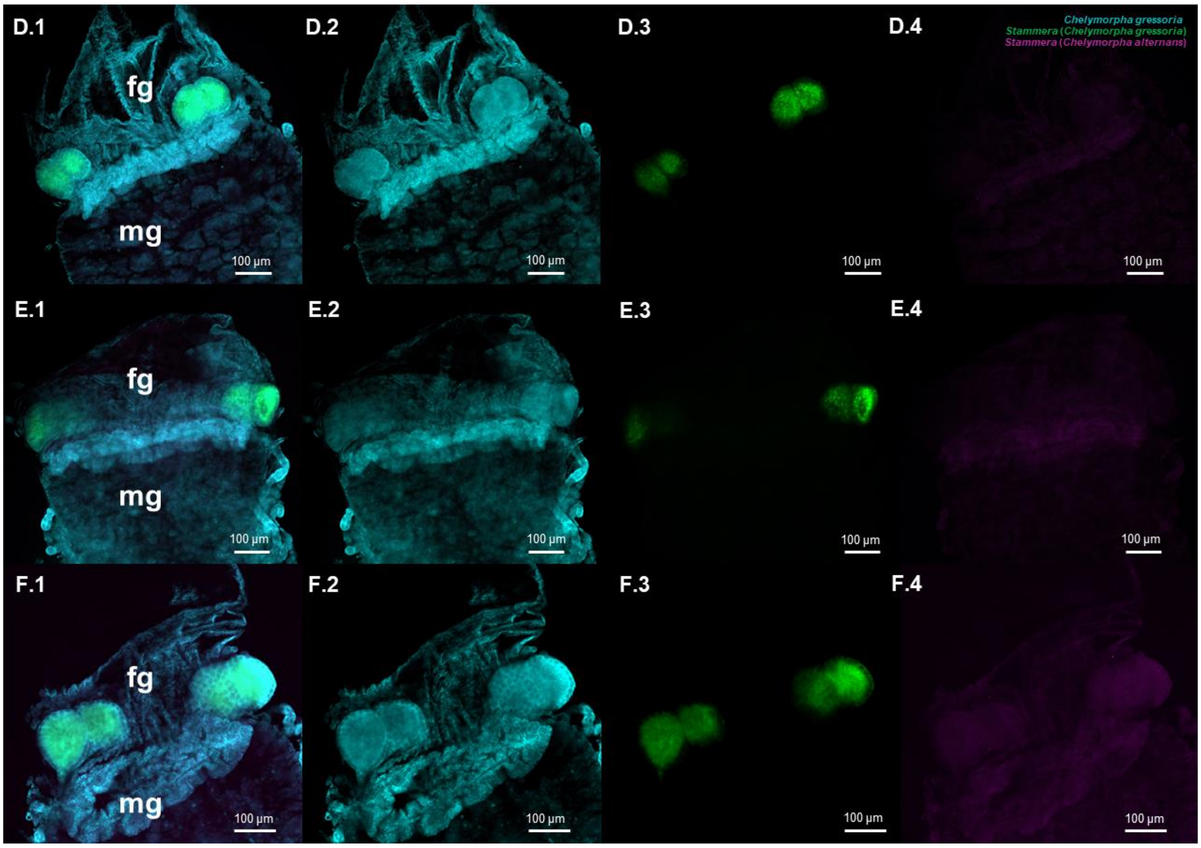

Figure S1iii (D-F)**.** Fluorescence *in situ* hybridization (FISH) replicates on whole-mounts of *Chelymorpha gressoria* larvae foregut symbiotic organs (untreated control) (*n* = 3 rep). Probes used: *Chelymorpha gressoria* host (cyan: 18S rRNA), *Stammera* from *Chelymorpha gressoria* (green: 16SrRNA), and *Stammera* from *Chelymorpha alternans* (magenta: 16S rRNA). **(1)** correspond to the merged channels images while the others correspond to individual channel images: **(2)** host probe, **(3)** *Stammera* from *Chelymorpha gressoria* probe, and **(4)** *Stammera* from *Chelymorpha alternans* probe. Abbreviations: fg, foregut; mg, midgut. Scale bars (100 µm) are included for reference.

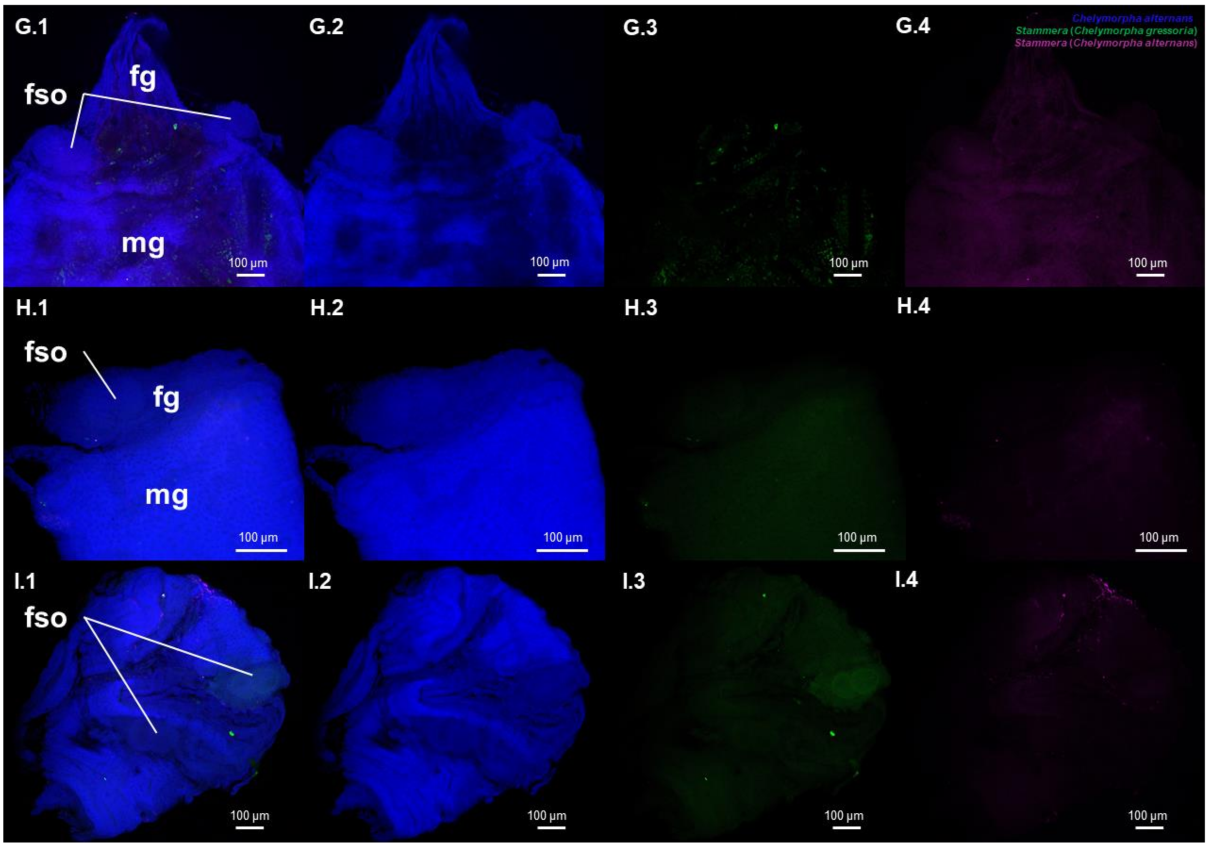

Figure S1iii (G-I)**.** Fluorescence *in situ* hybridization (FISH) replicates on whole-mounts of *Chelymorpha alternans* larvae foregut symbiotic organs (aposymbiotic) (*n* = 3 rep). Probes used: *Chelymorpha alternans* host (blue: 18S rRNA), *Stammera* from *Chelymorpha gressoria* (green: 16SrRNA), and *Stammera* from *Chelymorpha alternans* (magenta: 16S rRNA). **(1)** correspond to the merged channels images while the others correspond to individual channel images: **(2)** host probe, **(3)** *Stammera* from *Chelymorpha gressoria* probe, and **(4)** *Stammera* from *Chelymorpha alternans* probe. Abbreviations: fg, foregut; mg, midgut; fso, foregut symbiotic organs. Scale bars (100 µm) are included for reference.

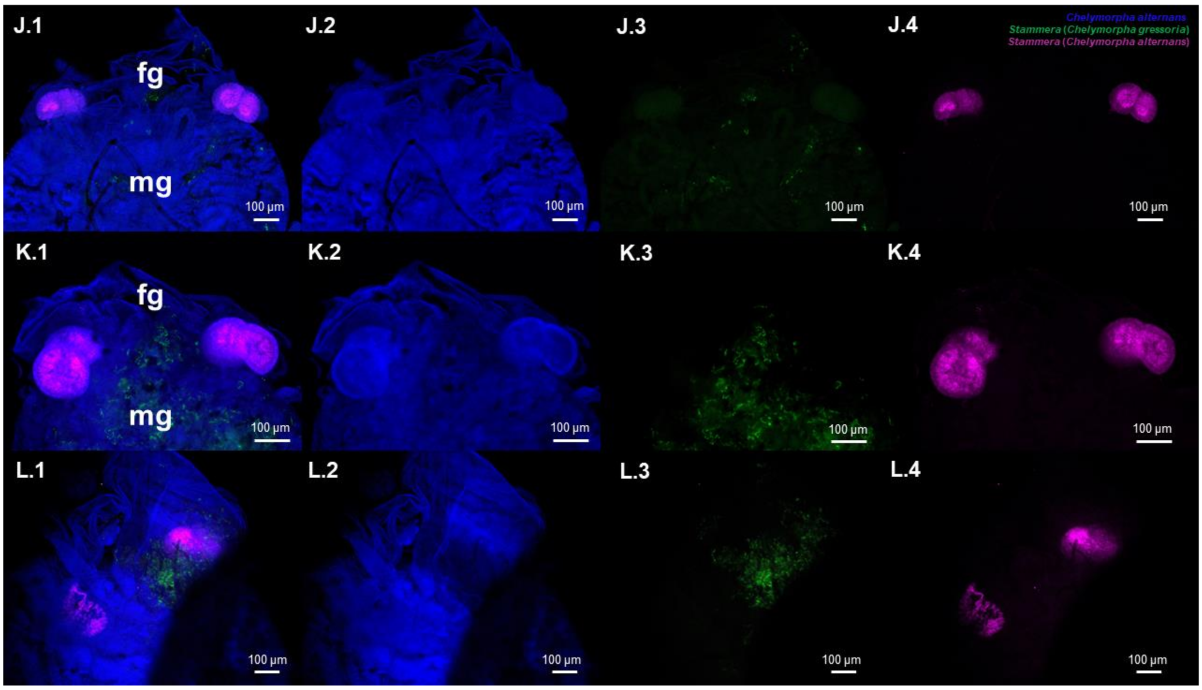

Figure S1iii (J-L)**.** Fluorescence *in situ* hybridization (FISH) replicates on whole-mounts of *Chelymorpha alternans* larvae foregut symbiotic organs (re-infected) (*n* = 3 rep). Probes used: *Chelymorpha alternans* host (blue: 18S rRNA), *Stammera* from *Chelymorpha gressoria* (green: 16SrRNA), and *Stammera* from *Chelymorpha alternans* (magenta: 16S rRNA). **(1)** correspond to the merged channels images while the others correspond to individual channel images: **(2)** host probe, **(3)** *Stammera* from *Chelymorpha gressoria* probe, and **(4)** *Stammera* from *Chelymorpha alternans* probe. Abbreviations: fg, foregut; mg, midgut. Scale bars (100 µm) are included for reference.

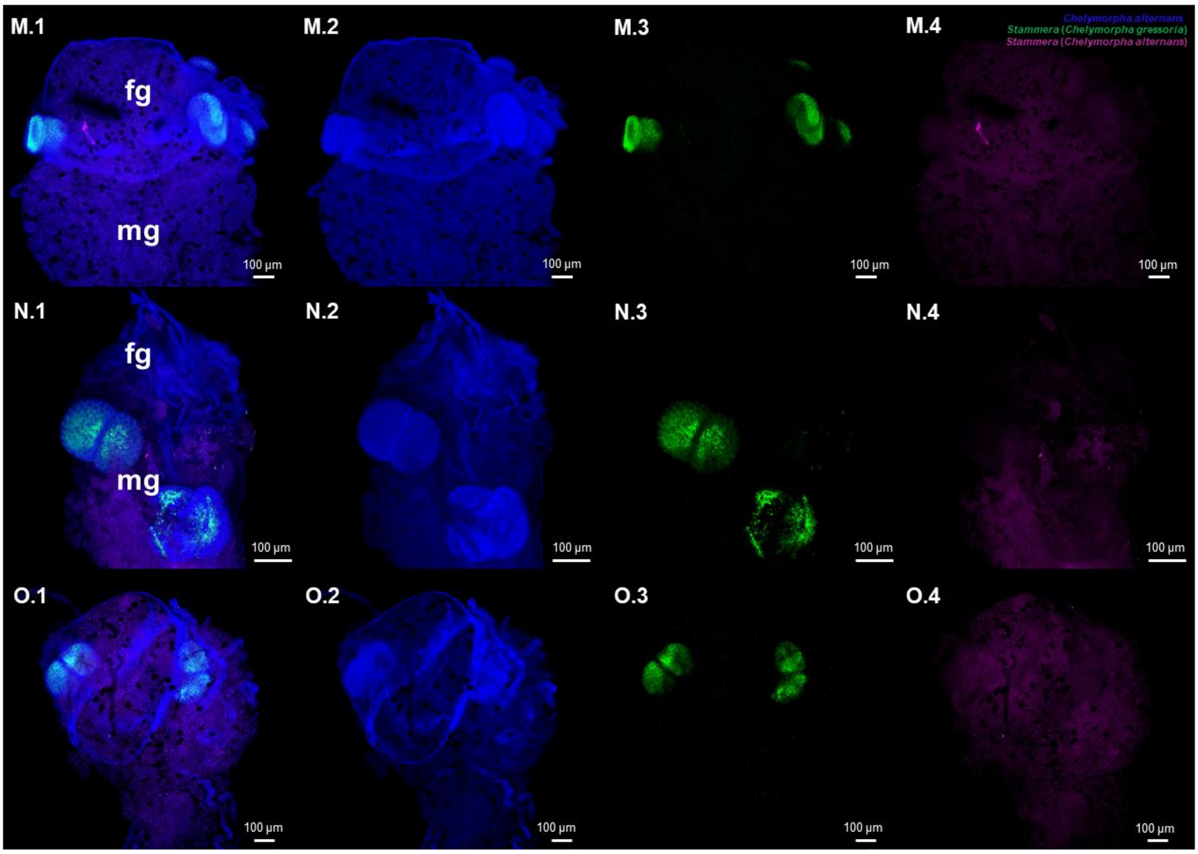

Figure S1iii (M-O)**.** Fluorescence *in situ* hybridization (FISH) replicates on whole-mounts of *Chelymorpha alternans* larvae foregut symbiotic organs (cross-infected) (*n* = 3 rep). Probes used: *Chelymorpha alternans* host (blue: 18S rRNA), *Stammera* from *Chelymorpha gressoria* (green: 16SrRNA), and *Stammera* from *Chelymorpha alternans* (magenta: 16S rRNA). **(1)** correspond to the merged channels images while the others correspond to individual channel images: **(2)** host probe, **(3)** *Stammera* from *Chelymorpha gressoria* probe, and **(4)** *Stammera* from *Chelymorpha alternans* probe. Abbreviations: fg, foregut; mg, midgut. Scale bars (100 µm) are included for reference.

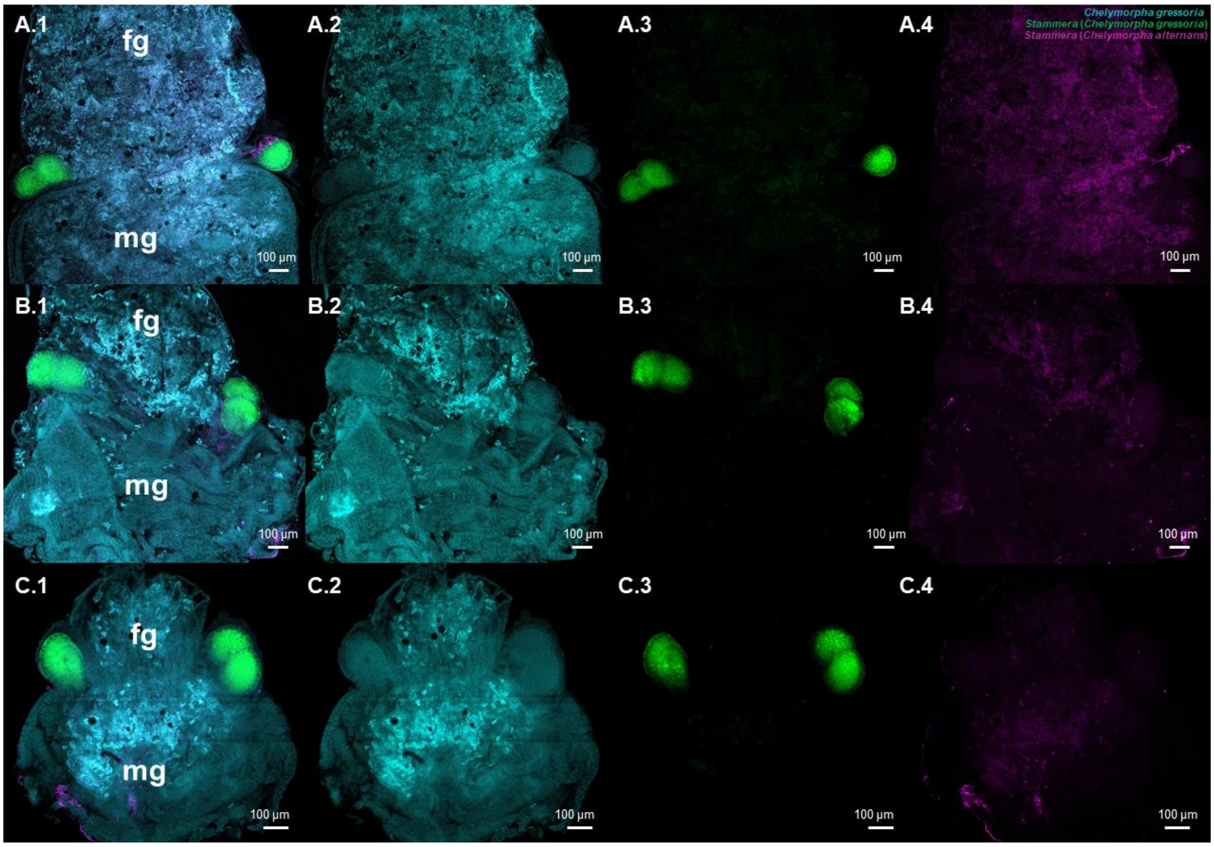

Figure S1iv (A-C)**.** Fluorescence *in situ* hybridization (FISH) replicates on whole-mounts of *Chelymorpha gressoria* larvae foregut symbiotic organs (untreated control) (*n* = 3 rep). Probes used: *Chelymorpha gressoria* host (cyan: 18S rRNA), *Stammera* from *Chelymorpha gressoria* (green: 16SrRNA), and *Stammera* from *Chelymorpha alternans* (magenta: 16S rRNA). **(1)** correspond to the merged channels images while the others correspond to individual channel images: **(2)** host probe, **(3)** *Stammera* from *Chelymorpha gressoria* probe, and **(4)** *Stammera* from *Chelymorpha alternans* probe. Abbreviations: fg, foregut; mg, midgut. Scale bars (100 µm) are included for reference.

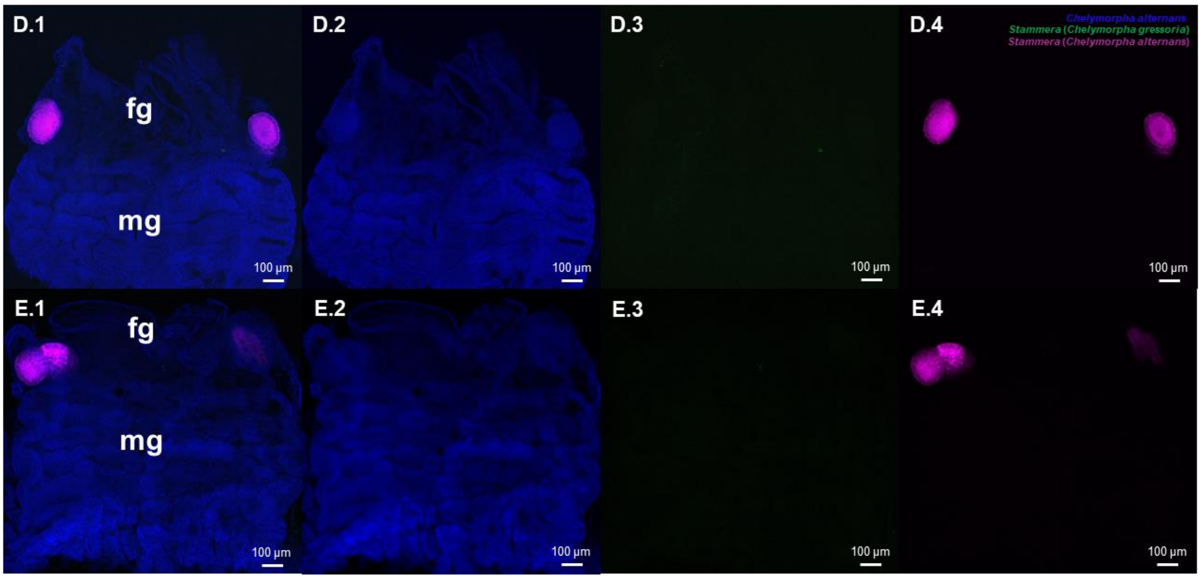

Figure S1iv (D-E)**.** Fluorescence *in situ* hybridization (FISH) replicates on whole-mounts of *Chelymorpha alternans* larvae foregut symbiotic organs (untreated control) (*n* = 2 rep). Probes used: *Chelymorpha alternans* host (blue: 18S rRNA), *Stammera* from *Chelymorpha gressoria* (green: 16SrRNA), and *Stammera* from *Chelymorpha alternans* (magenta: 16S rRNA). **(1)** correspond to the merged channels images while the others correspond to individual channel images: **(2)** host probe, **(3)** *Stammera* from *Chelymorpha gressoria* probe, and **(4)** *Stammera* from *Chelymorpha alternans* probe. Abbreviations: fg, foregut; mg, midgut. Scale bars (100 µm) are included for reference.

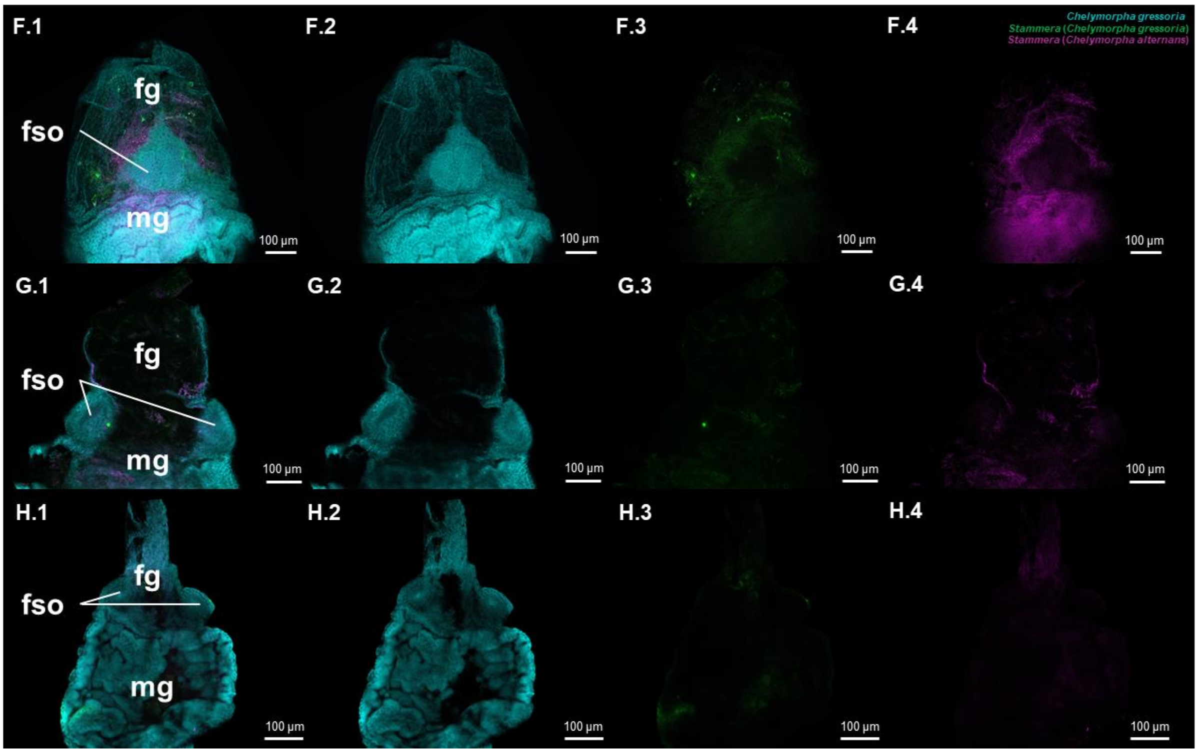

Figure S1iv (F-H)**.** Fluorescence *in situ* hybridization (FISH) replicates on whole-mounts of *Chelymorpha gressoria* larvae foregut symbiotic organs (aposymbiotic) (*n* = 3 rep). Probes used: *Chelymorpha gressoria* host (cyan: 18S rRNA), *Stammera* from *Chelymorpha gressoria* (green: 16SrRNA), and *Stammera* from *Chelymorpha alternans* (magenta: 16S rRNA). **(1)** correspond to the merged channels images while the others correspond to individual channel images: **(2)** host probe, **(3)** *Stammera* from *Chelymorpha gressoria* probe, and **(4)** *Stammera* from *Chelymorpha alternans* probe. Abbreviations: fg, foregut; mg, midgut; fso, foregut symbiotic organs. Scale bars (100 µm) are included for reference.

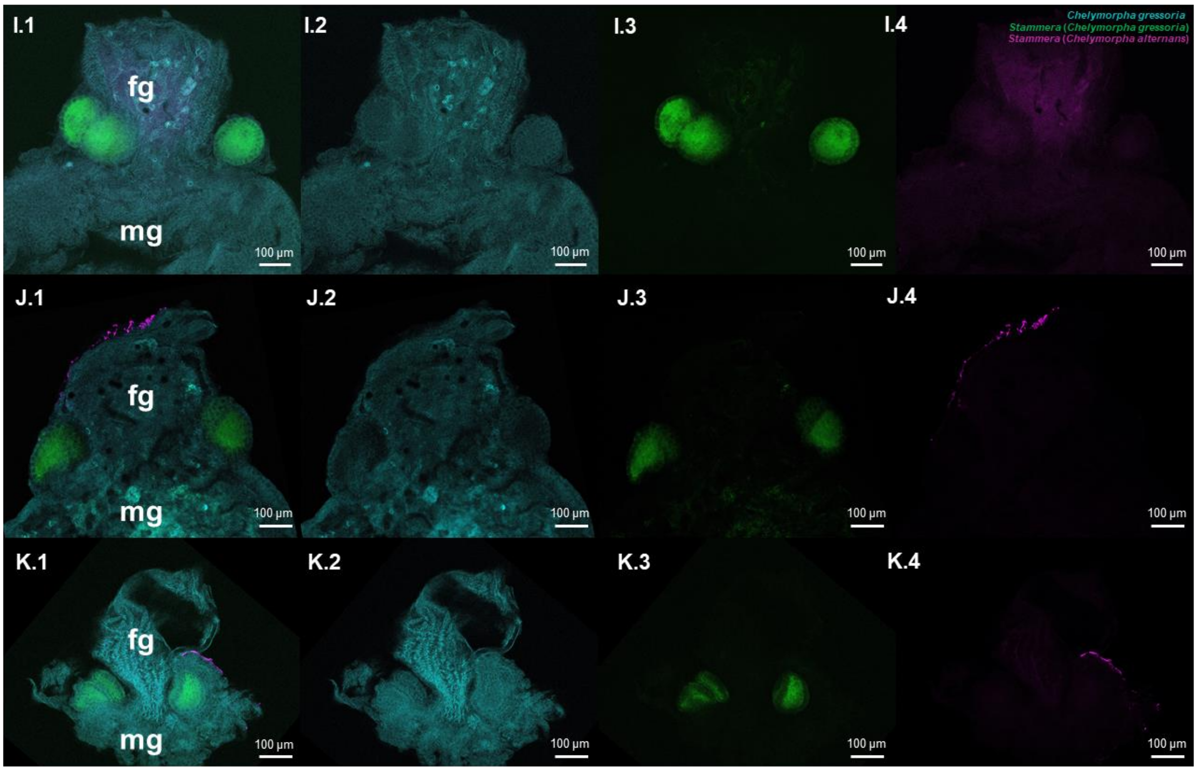

Figure S1iv (I-K)**.** Fluorescence *in situ* hybridization (FISH) replicates on whole-mounts of *Chelymorpha gressoria* larvae foregut symbiotic organs (re-infected) (*n* = 3 rep). Probes used: *Chelymorpha gressoria* host (cyan: 18S rRNA), *Stammera* from *Chelymorpha gressoria* (green: 16SrRNA), and *Stammera* from *Chelymorpha alternans* (magenta: 16S rRNA). **(1)** correspond to the merged channels images while the others correspond to individual channel images: **(2)** host probe, **(3)** *Stammera* from *Chelymorpha gressoria* probe, and **(4)** *Stammera* from *Chelymorpha alternans* probe. Abbreviations: fg, foregut; mg, midgut. Scale bars (100 µm) are included for reference.

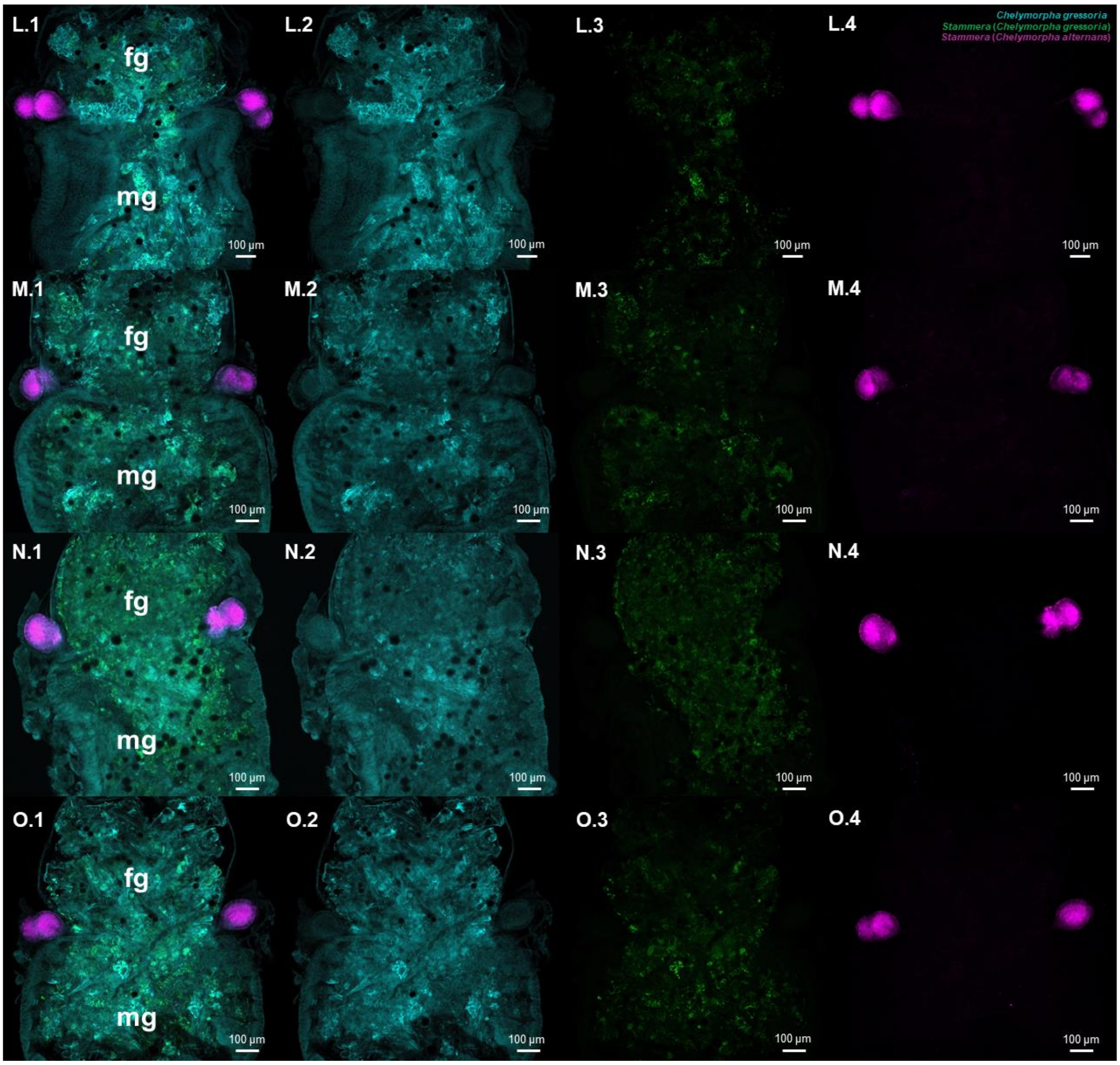

Figure S1iv (L-O)**.** Fluorescence *in situ* hybridization (FISH) replicates on whole-mounts of *Chelymorpha gressoria* larvae foregut symbiotic organs (cross-infected) (*n* = 4 rep). Probes used: *Chelymorpha gressoria* host (cyan: 18S rRNA), *Stammera* from *Chelymorpha gressoria* (green: 16SrRNA), and *Stammera* from *Chelymorpha alternans* (magenta: 16S rRNA). **(1)** correspond to the merged channels images while the others correspond to individual channel images: **(2)** host probe, **(3)** *Stammera* from *Chelymorpha gressoria* probe, and **(4)** *Stammera* from *Chelymorpha alternans* probe. Abbreviations: fg, foregut; mg, midgut. Scale bars (100 µm) are included for reference.

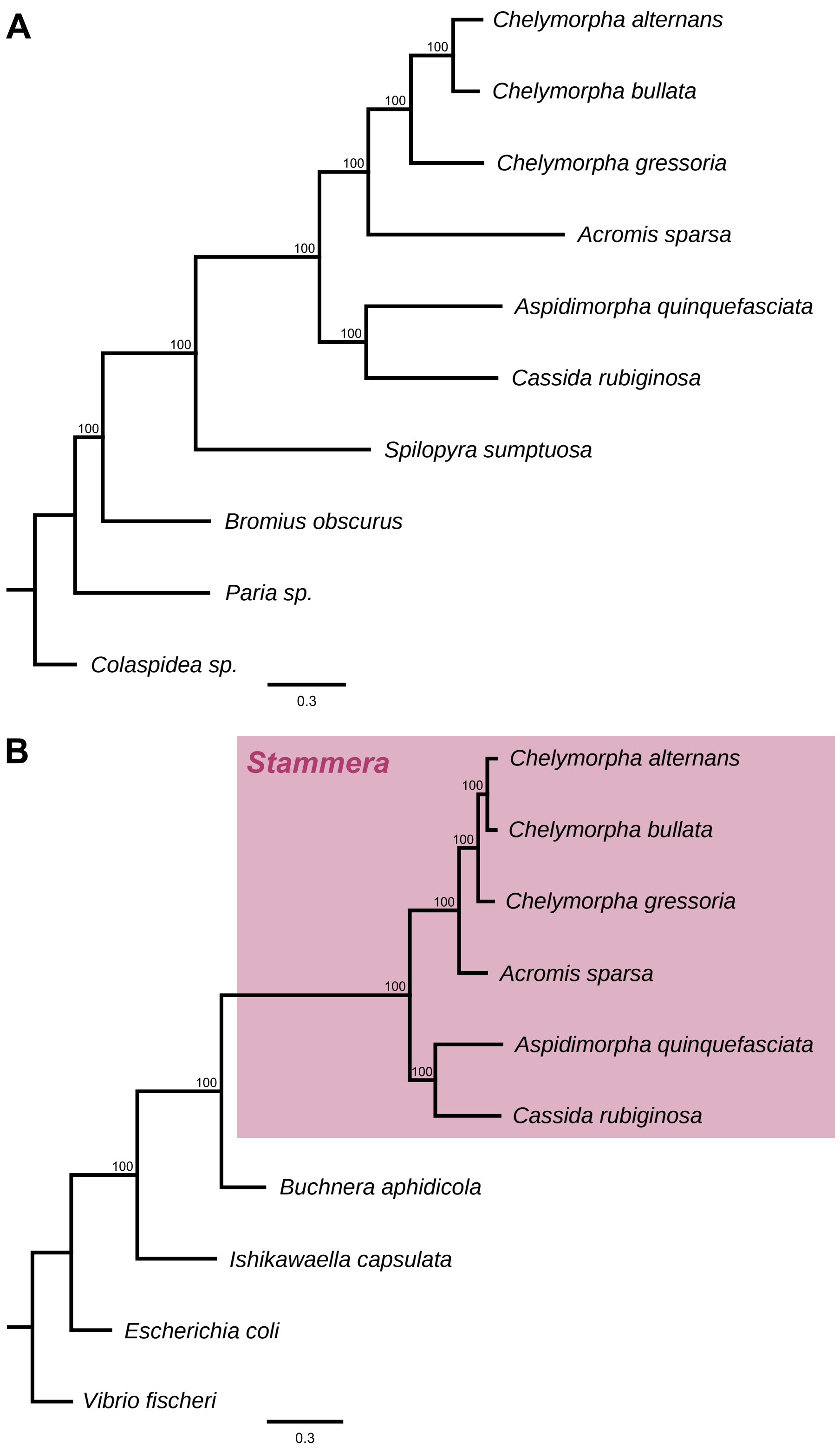

Figure S2. (A) Detailed host phylogeny including outgroups based on 15 mitochondrial genes. The phylogenetic tree was constructed by maximum likelihood (ML) methods. **(B)** Detailed *Stammera* phylogeny including outgroups based on maximum likelihood (ML) methods. The phylogenomic tree was constructed based on a concatenated alignment of 61 single-copy core genes in RAxML, using the most appropriate substitution model according to PartitionFinder2. Bootstrap support values are shown for each node.

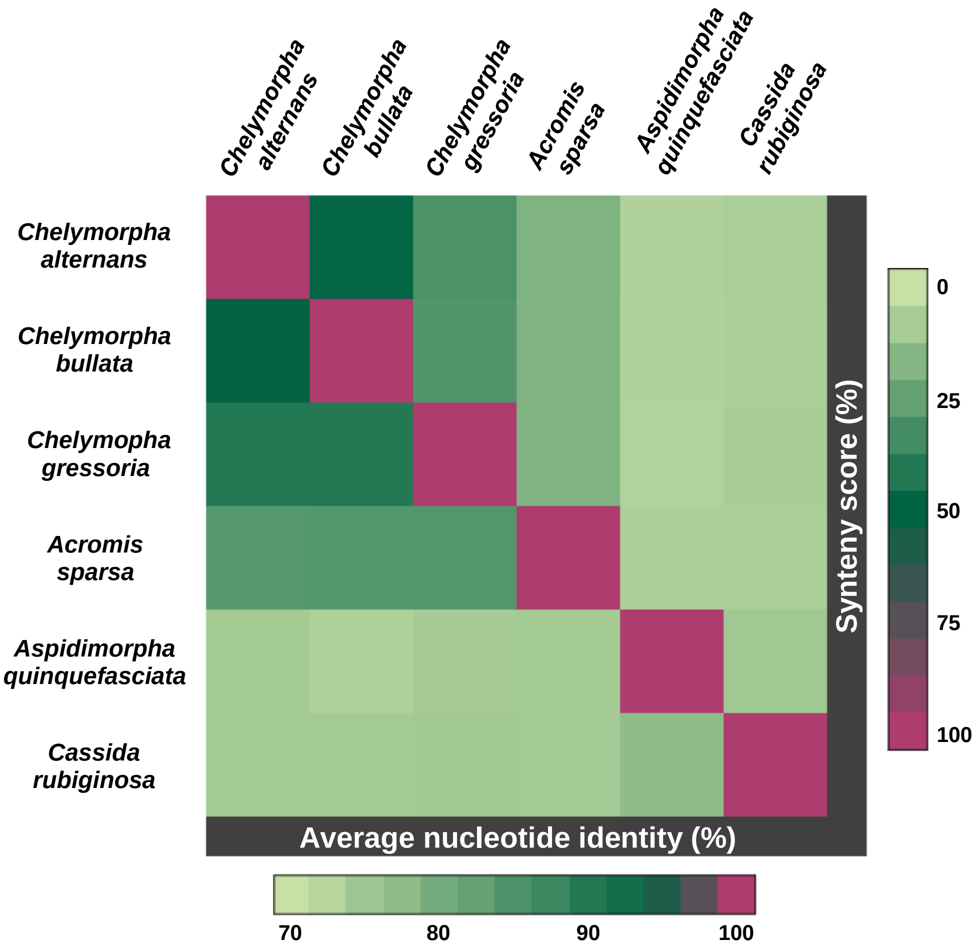

Figure S3. Heatmap illustrating genetic distances between *Stammera* symbionts. Pairwise comparisons of average nucleotide identity (ANI) (light green, 70%; dark green, 95%) and synteny scores (light green, 0%; dark green, 60%) are shown. Source data are provided as a Source Data file.

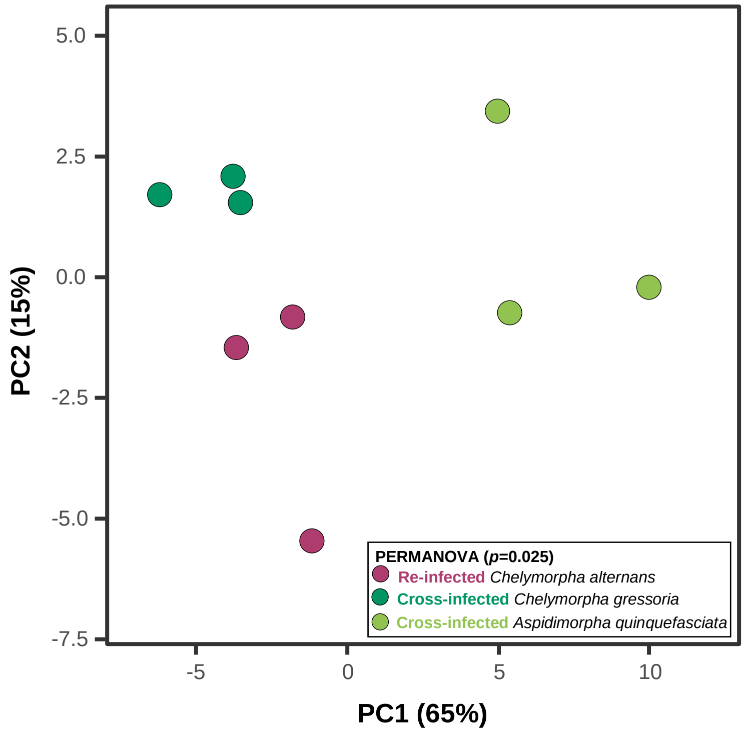

Figure S4. Principal coordinate analysis (PCA) of host differentially expressed genes after colonization of foregut symbiotic organs by *Stammera* symbionts (n = 3 replicates per treatment). Significant clustering was assessed by PERMANOVA (after removing the batch effects) based on Euclidean distances between samples (p = 0.025, Supplementary Data 4A).

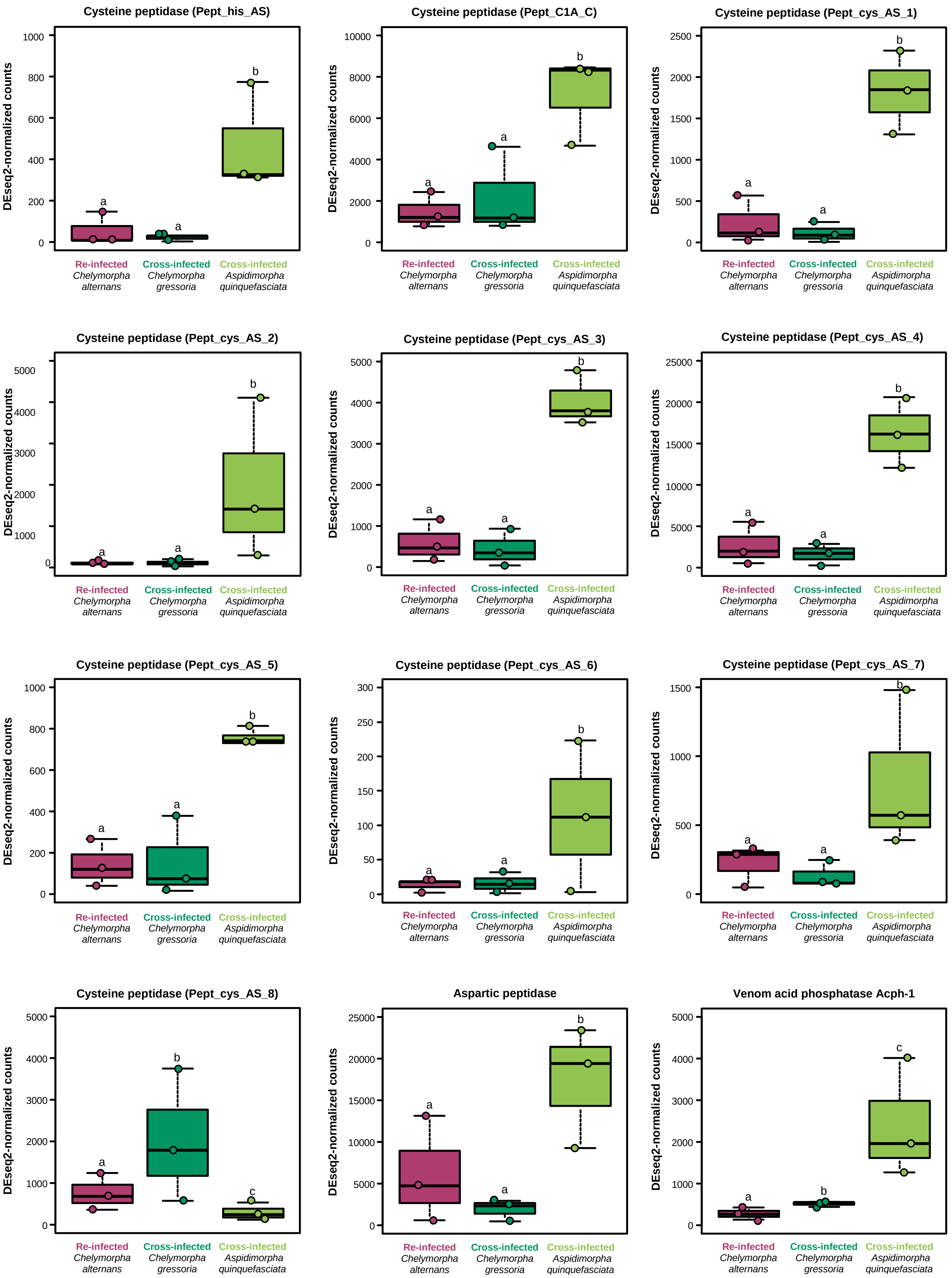

Cysteine peptidase (Pept_his_AS)
Cysteine peptidase (Pept_C1A_C)
Cysteine peptidase (Pept_cys_AS_1)
Cysteine peptidase (Pept_cys_AS_2)
Cysteine peptidase (Pept_cys_AS_3)
Cysteine peptidase (Pept_cys_AS_4)
Cysteine peptidase (Pept_cys_AS_5)
Cysteine peptidase (Pept_cys_AS_6)
Cysteine peptidase (Pept_cys_AS_7)
Cysteine peptidase (Pept_cys_AS_8)
Aspartic peptidase
Venom acid phosphatase Acph-1
DEseq2-normalized counts
Re-infected Chelymorpha alternans
Cross-infected Chelymorpha gressoria
Cross-infected Aspidimorpha quinquefasciata
a
b
c

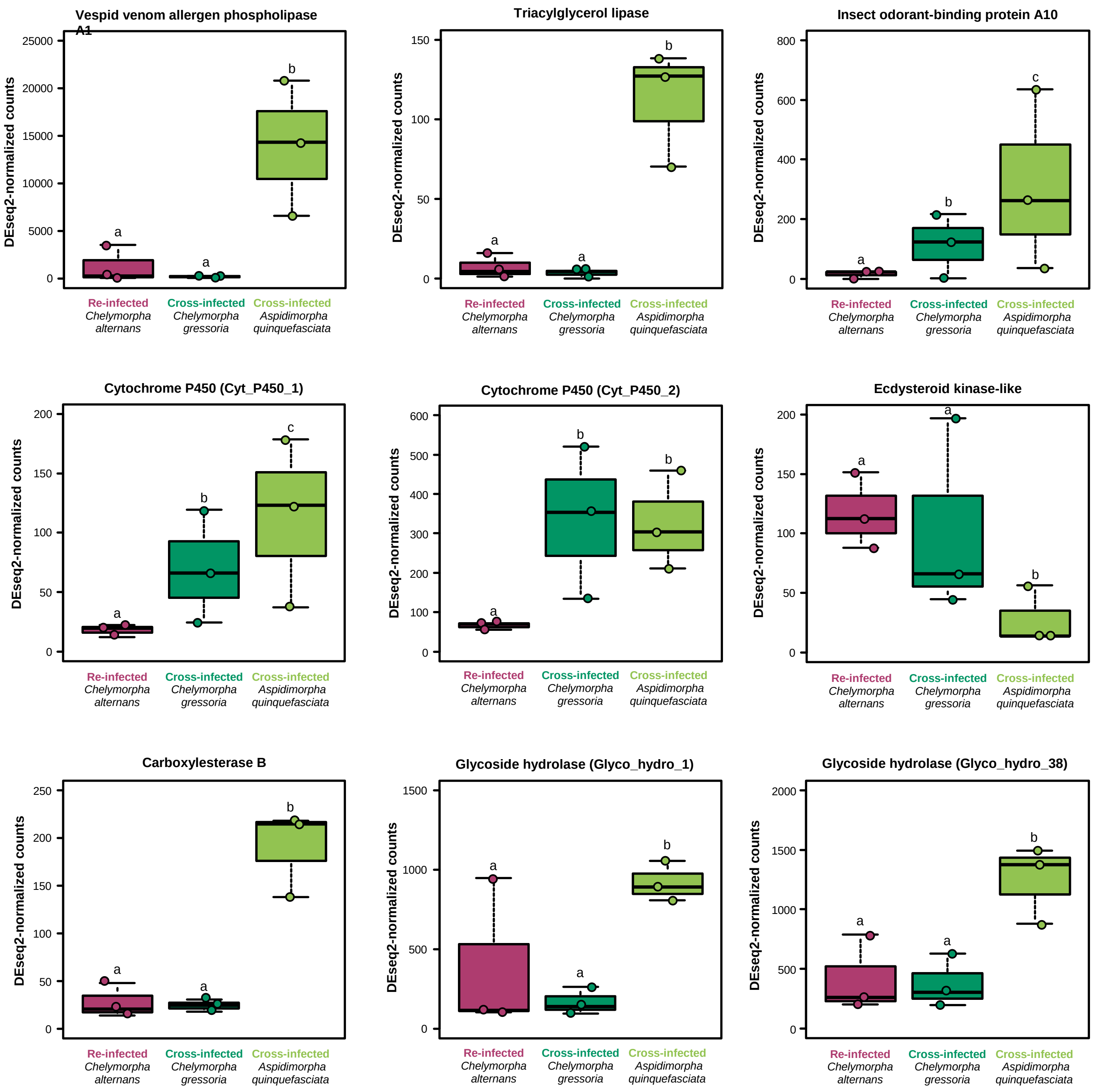

Figure S5. Differential expression of host genes after colonization of foregut symbiotic organs by the native symbiont (magenta), non-native symbiont of *Chelymorpha gressoria* (dark green), or non-native symbiont of *Aspidimorpha quinquefasciata* (light green). Counts were normalized by DESeq2's median of ratios. Lines represent medians, boxes indicate 25-75 percentiles, whiskers denote range, and dots indicate replicates (*n* = 3). Different letters indicated significant differences (See Supplementary Data 4B). Source data are provided as a Source Data file.

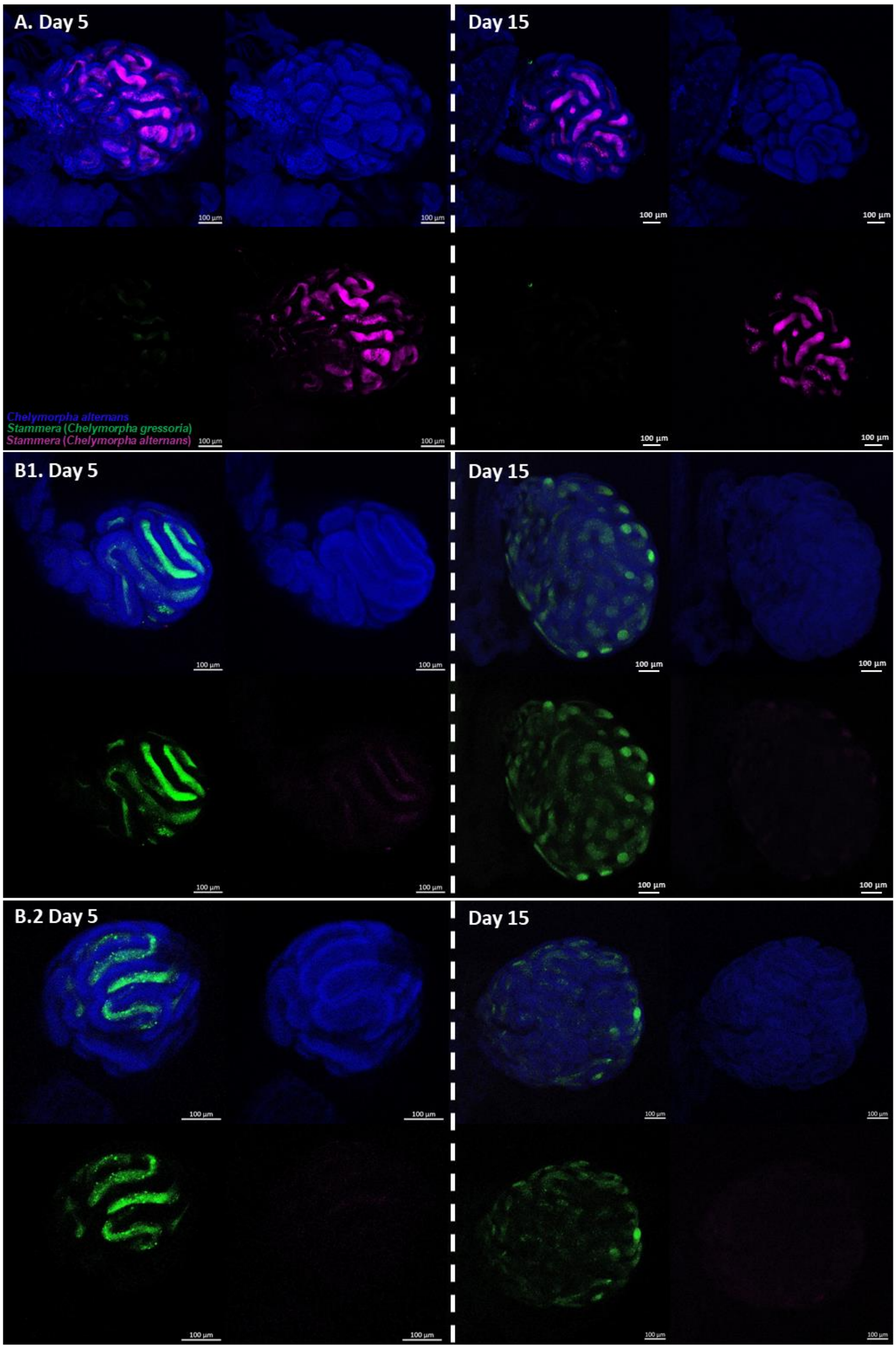

Figure S6. Fluorescence *in situ* hybridization (FISH) on whole-mounts of ovary-associated glands from *Chelymorpha alternans* females **(A)** re-infected with native symbiont or **(B)** cross-infected with non-native symbiont at 5 and 15 days after emergence. Each image is from a single individual and is shown for illustrative purposes. Both glands of the cross-infected sample were examined. Probes used: *Chelymorpha alternans* host (blue: 18S rRNA), *Stammera* from *Chelymorpha gressoria* (green: 16SrRNA), and *Stammera* from *Chelymorpha alternans* (magenta: 16S rRNA). The top left image corresponds to the merged channels images while the others correspond to individual channel images: top right image for the host probe, bottom left image for the probe targeting *Stammera* from *Chelymorpha gressoria*, and bottom right image for the probe targeting *Stammera* from *Chelymorpha alternans*. Scale bars (100 µm) are included for reference.

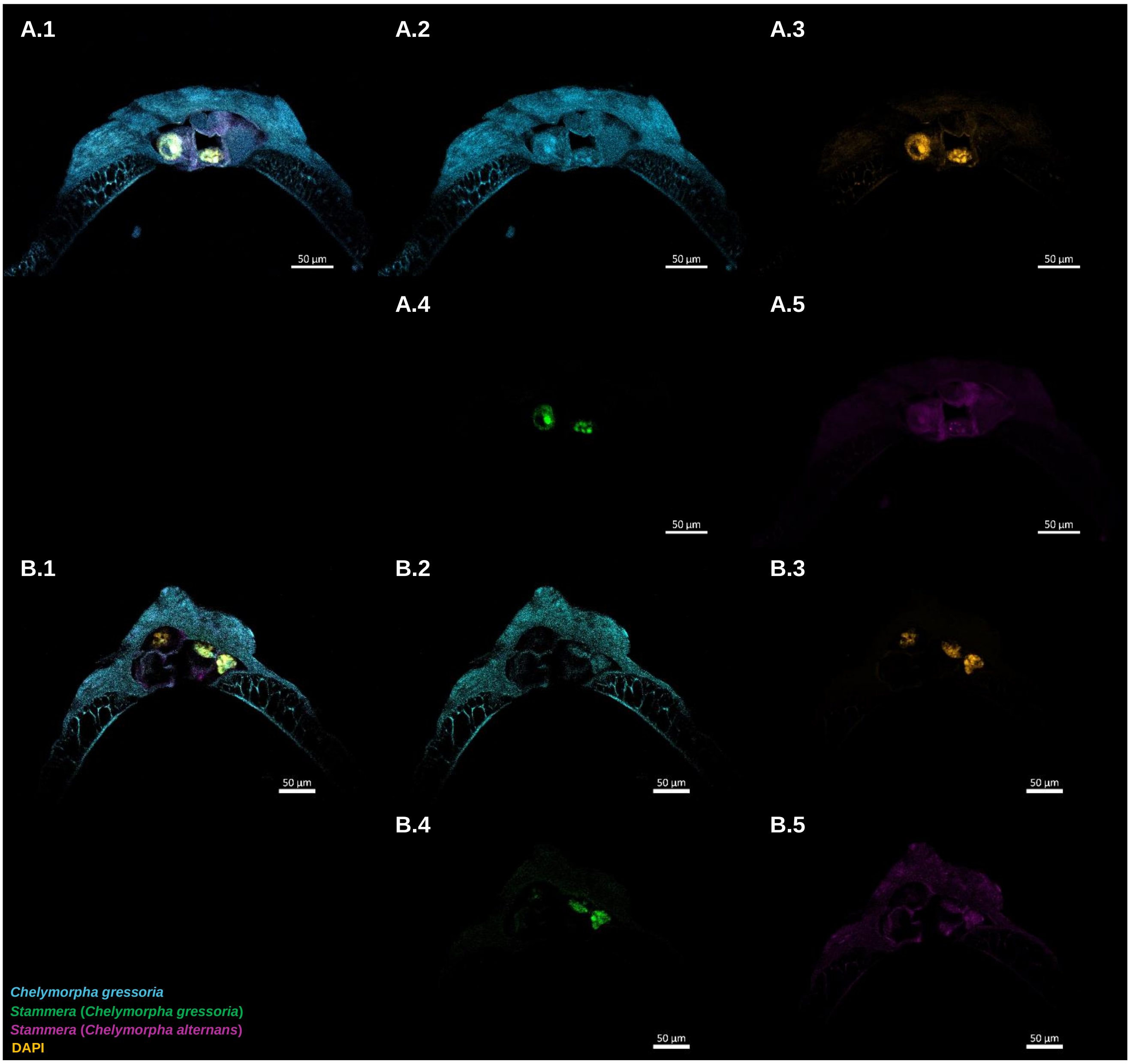

Figure S7. (A-B) Fluorescence *in situ* hybridization (FISH) replicates on longitudinal sections of eggs laid by *Chelymorpha gressoria* females (untreated control) (*n* = 2 rep). Probes used: *Chelymorpha gressoria* host (cyan: 18S rRNA), the DAPI-stained DNA (orange), *Stammera* from *Chelymorpha gressoria* (green: 16SrRNA), and *Stammera* from *Chelymorpha alternans* (magenta: 16S rRNA). Autofluorescence is observed for egg shells and caplets. **(1)** correspond to the merged channels images while the others correspond to individual channel images: **(2)** host probe, **(3)** DAPI-stained DNA, **(4)** *Stammera* from *Chelymorpha gressoria* probe, and **(5)** *Stammera* from *Chelymorpha alternans* probe. Scale bars (50 µm) are included for reference.

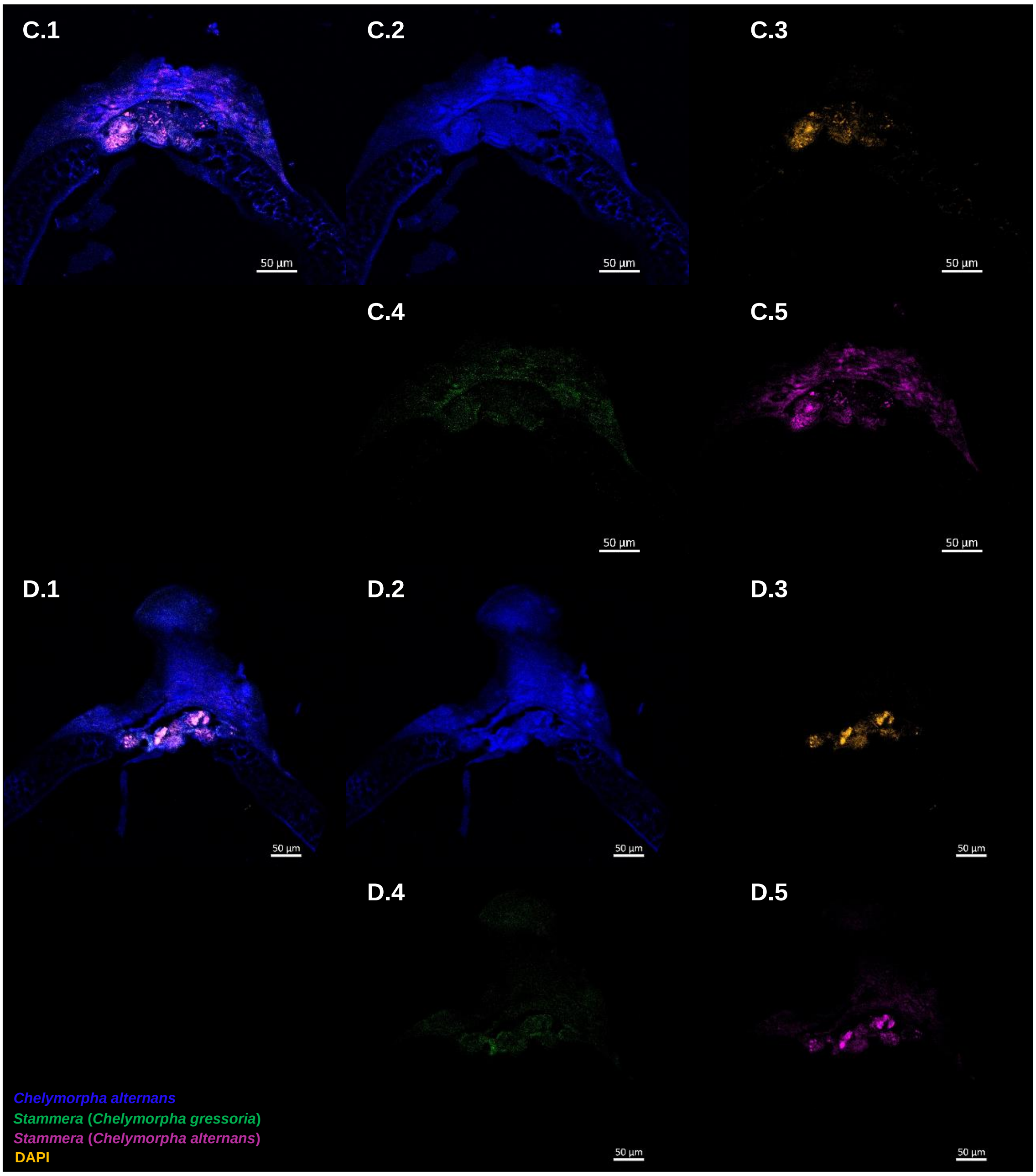

Figure S7. (C-D) Fluorescence *in situ* hybridization (FISH) replicates on longitudinal sections of eggs laid by *Chelymorpha alternans* females (untreated control) (*n* = 2 rep). Probes used: *Chelymorpha alternans* host (blue: 18S rRNA), the DAPI-stained DNA (orange), *Stammera* from *Chelymorpha gressoria* (green: 16SrRNA), and *Stammera* from *Chelymorpha alternans* (magenta: 16S rRNA). Autofluorescence is observed for egg shells and caplets. **(1)** correspond to the merged channels images while the others correspond to individual channel images: **(2)** host probe, **(3)** DAPI-stained DNA, **(4)** *Stammera* from *Chelymorpha gressoria* probe, and **(5)** *Stammera* from *Chelymorpha alternans* probe. Scale bars (50 µm) are included for reference.

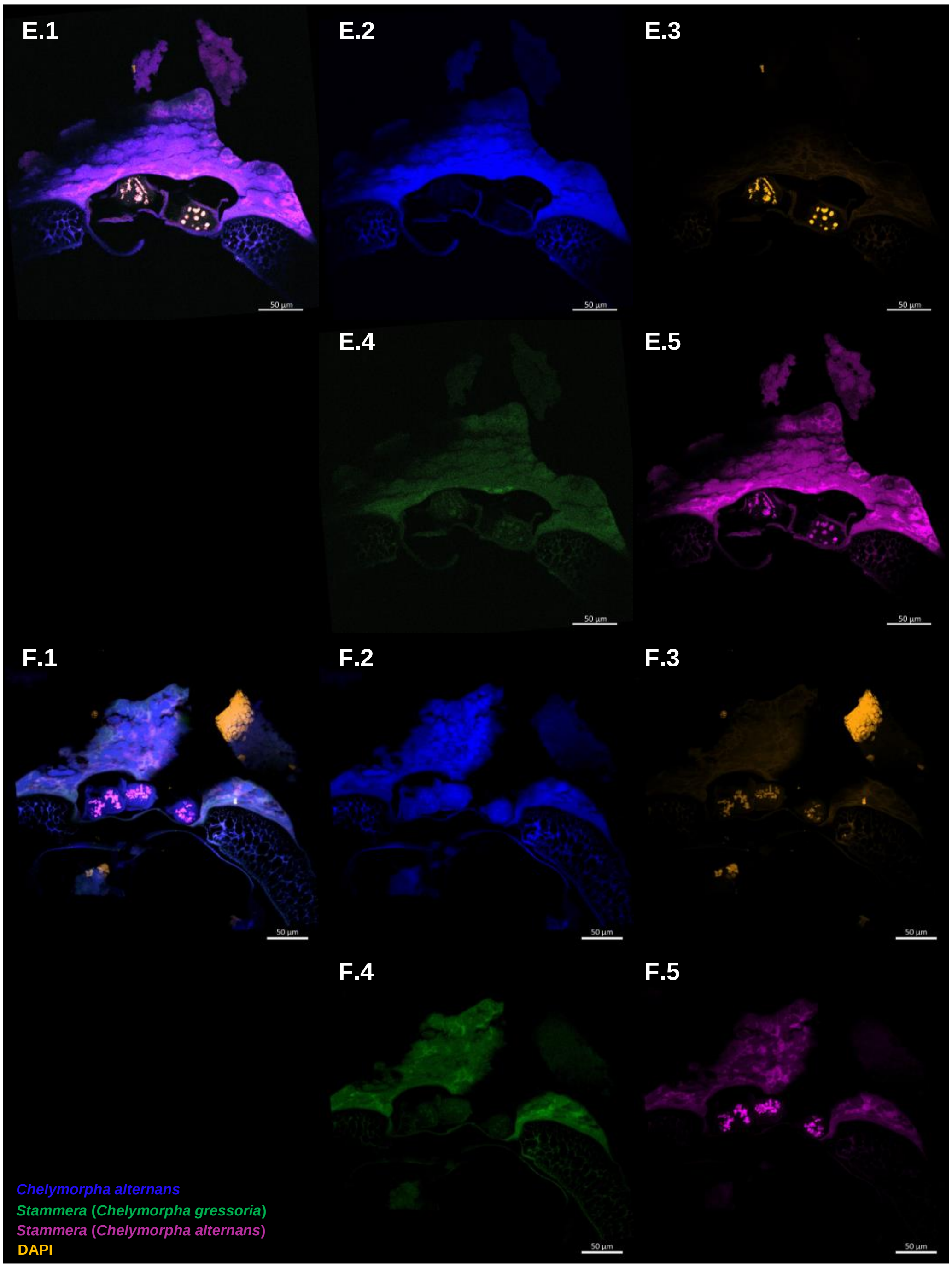

Figure S7. (E-F) Fluorescence *in situ* hybridization (FISH) replicates on longitudinal sections of eggs laid by *Chelymorpha alternans* females re-infected by the native symbiont (*n* = 2 rep). Probes used: *Chelymorpha alternans* host (blue: 18S rRNA), the DAPI-stained DNA (orange), *Stammera* from *Chelymorpha gressoria* (green: 16SrRNA), and *Stammera* from *Chelymorpha alternans* (magenta: 16S rRNA). Autofluorescence is observed for egg shells and caplets. **(1)** correspond to the merged channels images while the others correspond to individual channel images: **(2)** host probe, **(3)** DAPI-stained DNA, **(4)** *Stammera* from *Chelymorpha gressoria* probe, and **(5)** *Stammera* from *Chelymorpha alternans* probe. Scale bars (50 µm) are included for reference.

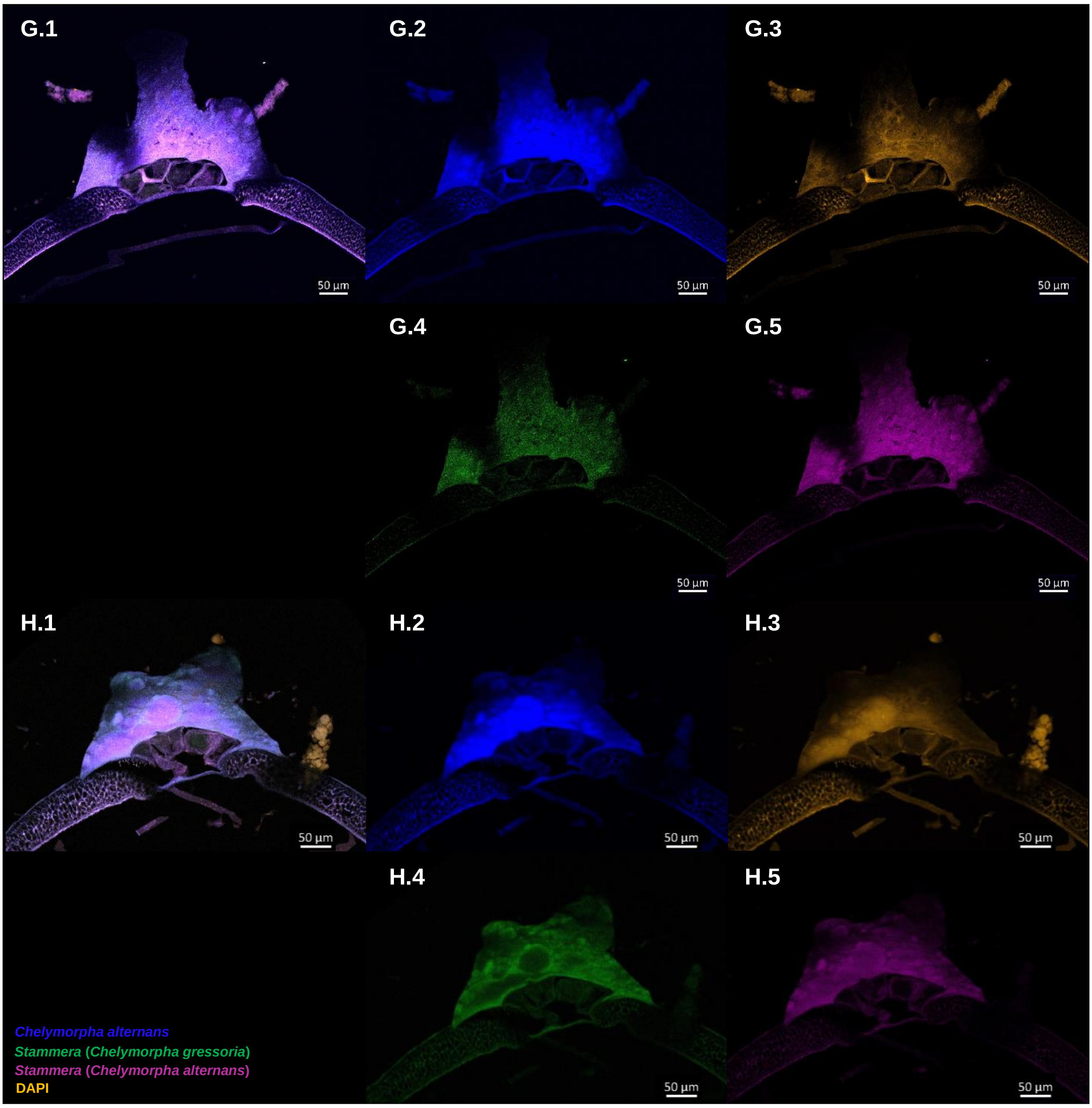
G.1
G.2
G.3
G.4
G.5
H.1
H.2
H.3
H.4
H.5
50 µm
Chelymorpha alternans
Stammera (Chelymorpha gressoria)
Stammera (Chelymorpha alternans)
DAPI

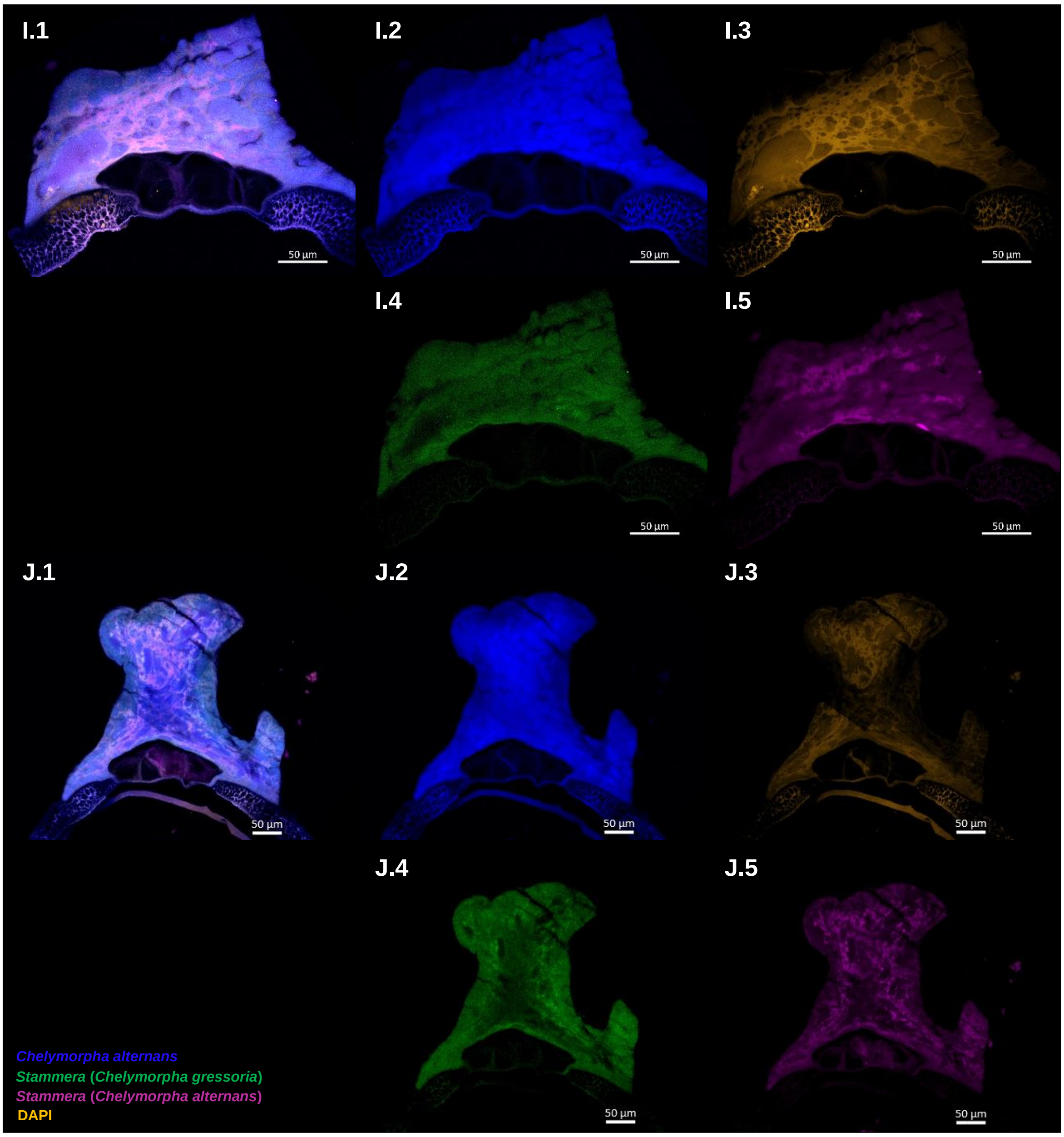
I.1
I.2
I.3
I.4
I.5
J.1
J.2
J.3
J.4
J.5
50 µm
Chelymorpha alternans
Stammera (Chelymorpha gressoria)
Stammera (Chelymorpha alternans)
DAPI

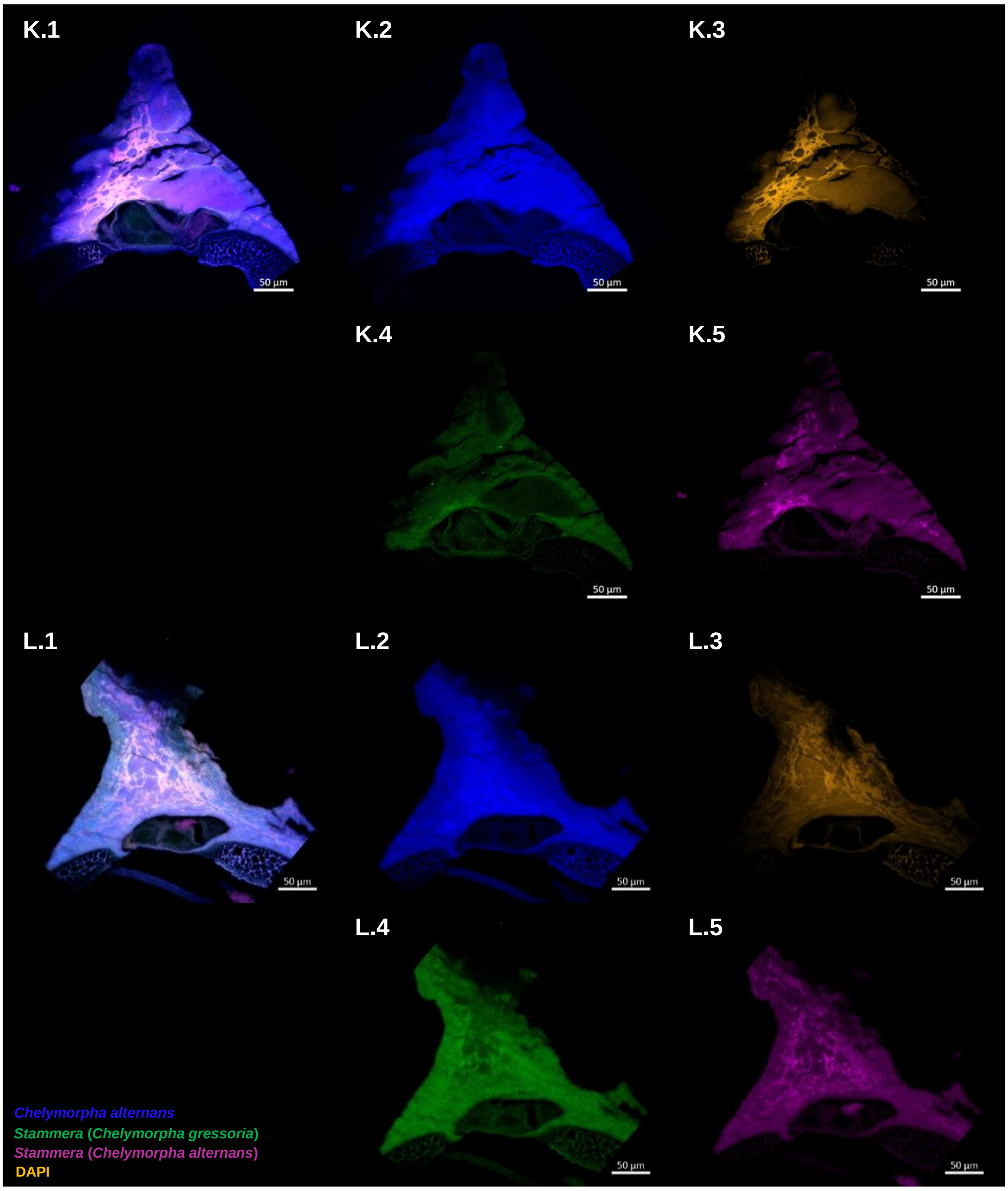

Figure S7. (G-L) Fluorescence *in situ* hybridization (FISH) replicates on longitudinal sections of eggs laid by *Chelymorpha alternans* females cross-infected by the non-native symbiont (*n* = 2 rep with 1-4 eggs per replicate). Probes used: *Chelymorpha alternans* host (blue: 18S rRNA), the DAPI-stained DNA (orange), *Stammera* from *Chelymorpha gressoria* (green: 16SrRNA), and *Stammera* from *Chelymorpha alternans* (magenta: 16S rRNA). Autofluorescence is observed for egg shells and caplets. **(1)** correspond to the merged channels images while the others correspond to individual channel images: **(2)** host probe, **(3)** DAPI-stained DNA, **(4)** *Stammera* from *Chelymorpha gressoria* probe, and **(5)** *Stammera* from *Chelymorpha alternans* probe. **(G-H)** correspond to the first replicate and **(I-L)** correspond to the second replicate. Scale bars (50 µm) are included for reference.

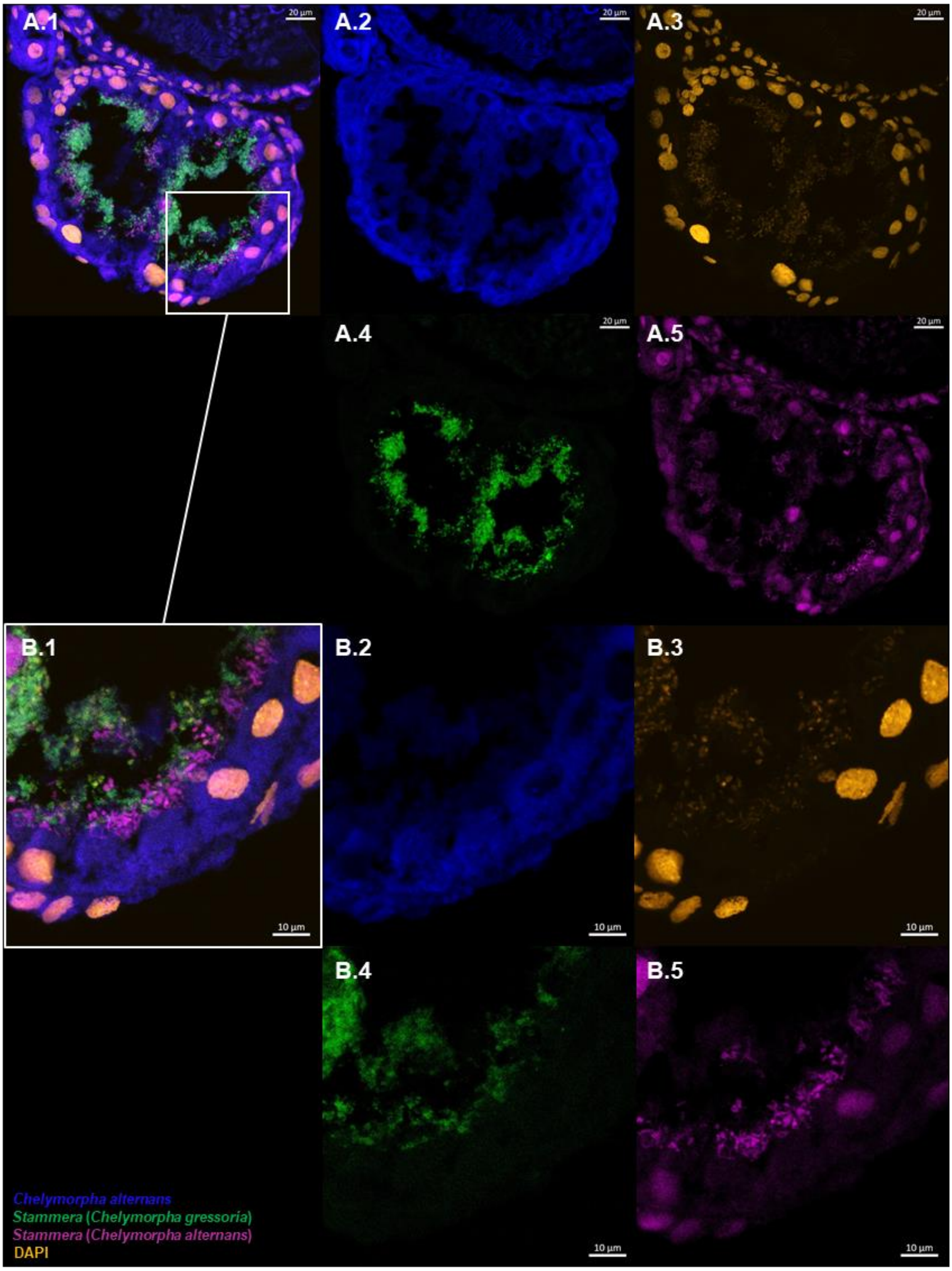

Figure S8. (A) Fluorescence *in situ* hybridization (FISH) cross-section of foregut symbiotic organs of 5-day-old, dually infected *Chelymorpha alternans* larvae. The image is from a single individual and is shown for illustrative purposes. **(B)** Close-up view of the foregut symbiotic organ. Probes used: *Chelymorpha alternans* host (blue: 18S rRNA), the DAPI-stained DNA (orange), *Stammera* from *Chelymorpha gressoria* (green: 16SrRNA), and *Stammera* from *Chelymorpha alternans* (magenta: 16S rRNA). **(1)** correspond to the merged channels images while the others correspond to individual channel images: **(2)** *Chelymorpha alternans* host probe, **(3)** DAPI-stained DNA, **(4)** *Stammera* from *Chelymorpha gressoria* probe, and **(5)** *Stammera* from *Chelymorpha alternans* probe. Scale bars are included for reference.

Host	Gene prediction	Pathway	Category
C. alternans	Serine--tRNA ligase (serS)	Aminoacyl-tRNA synthetases	Translation, ribosomal structure and biogenesis
C. alternans	Glyceraldehyde-3-phosphate dehydrogenase A (gapA)	Glycolysis	Carbohydrate transport and metabolism
C. alternans	Inner membrane protein YdjM (ydjM)	NA	General function prediction only
C. alternans	Flavodoxin (fldA)	Heme biosynthesis	Energy production and conversion
C. alternans	hypothetical protein	Unknown	Unknown
C. gressoria	Ribosomal RNA large subunit methyltransferase E (rlmE)	23S rRNA modification	Translation, ribosomal structure and biogenesis
C. gressoria	Serine hydroxymethyltransferase (glyA)	Serine biosynthesis	Amino acid transport and metabolism
C. gressoria	UDP-N-acetylglucosamine 1-carboxyvinyltransferase (murA)	Mureine biosynthesis	Cell wall/membrane/envelope biogenesis

Table S1. Comparative list of the only eight genes that differ between the symbionts of *Chelymorpha alternans* and *Chelymorpha gressoria*.
