# Supplementary material for: Fidelity in co-diversified symbiosis
Source: Nat Commun. 2026 Feb 12;17:1644. doi: 10.1038/s41467-026-69366-4 (PMC12905250; doi:10.1038/s41467-026-69366-4)
Supplement: Supplementary file 2 — Description of Additional Supplementary Files [file 41467_2026_69366_MOESM2_ESM.pdf]

Description of Additional Supplementary Files

Supplementary Data 1. Tortoise beetle species used in the present study.

Supplementary Data 2A. Statistical analyses of Figure 4A.

Supplementary Data 2B. Statistical analyses of Figure 4B.

Supplementary Data 2C. Statistical analyses of Figure 4C.

Supplementary Data 2D. Statistical analyses of Figures 4D-E.

Supplementary Data 3A. Number of reads in each library (input read pairs) and number and percentage of reads mapped to *Chelymorpha alternans* host and *Stammera* symbionts.

Supplementary Data 3B. DEseq2-normalized counts per treatment. The three replicates are represented for each treatment.

Supplementary Data 3C. Differentially expressed host genes after colonization of foregut symbiotic organs by the non-native symbiont of *Chelymorpha gressoria* relative to the native symbiont. The genes were considered significantly expressed at adjusted p-value < 0.05 and a fold-change > 2.

Supplementary Data 3D. Differentially expressed host genes after colonization of foregut symbiotic organs by the non-native symbiont of *Aspidimorpha quinquefasciata* relative to the native symbiont. The genes were considered significantly expressed at adjusted p-value < 0.05 and a fold-change > 2.

Supplementary Data 3E. Differentially expressed host genes after colonization of its foregut symbiotic organs by the non-native symbiont of *Aspidimorpha quinquefasciata* relative to the non-native symbiont of *Chelymorpha gressoria*. The genes were considered significantly expressed at adjusted p-value < 0.05 and a fold-change > 2.

Supplementary Data 3F. Presence and absence of differentially expressed genes per treatment. Genes shared between treatments can be identified.

Supplementary Data 4A. Statistical analyses of Figure S4.

Supplementary Data 4B. Statistical analyses of Figures 5D and S5.

Supplementary Data 4C. Statistical analyses of Figure 5E.

Supplementary Data 4D. Statistical analyses of Figure 5F.

Supplementary Data 4E. Statistical analyses of Figure 6.

Supplementary Data 4F. Statistical analyses of Figure 7C.

Supplementary Data 4G. Statistical analyses of Figure 7E.

Supplementary Data 5A. FISH probes used in the study.

Supplementary Data 5B. Accession numbers to reconstruct the host and symbiont phylogenies.

Supplementary Data 5C. Diagnostic primer pairs used for the specific detection of *Stammera* from tortoise beetles and for positive control amplification of host DNA.

Supplementary Data 5D. Primer pairs used for qPCR assays.
