# Supplementary material for: Fidelity in co-diversified symbiosis
Source: Nat Commun. 2026 Feb 12;17:1644. doi: 10.1038/s41467-026-69366-4 (PMC12905250; doi:10.1038/s41467-026-69366-4)
Supplement: Supplementary file 8 — Reporting Summary [file 41467_2026_69366_MOESM8_ESM.pdf]

nature portfolio

Corresponding author(s): Ines Pons and Hassan Salem

Last updated by author(s): Dec 25, 2025

Reporting Summary

Nature Portfolio wishes to improve the reproducibility of the work that we publish. This form provides structure for consistency and transparency in reporting. For further information on Nature Portfolio policies, see our Editorial Policies and the Editorial Policy Checklist.

Statistics

For all statistical analyses, confirm that the following items are present in the figure legend, table legend, main text, or Methods section.

n/a	Confirmed	
☐	☒	The exact sample size (*n*) for each experimental group/condition, given as a discrete number and unit of measurement
☐	☒	A statement on whether measurements were taken from distinct samples or whether the same sample was measured repeatedly
☐	☒	The statistical test(s) used AND whether they are one- or two-sided<br>*Only common tests should be described solely by name; describe more complex techniques in the Methods section.*
☐	☒	A description of all covariates tested
☐	☒	A description of any assumptions or corrections, such as tests of normality and adjustment for multiple comparisons
☐	☒	A full description of the statistical parameters including central tendency (e.g. means) or other basic estimates (e.g. regression coefficient) AND variation (e.g. standard deviation) or associated estimates of uncertainty (e.g. confidence intervals)
☐	☒	For null hypothesis testing, the test statistic (e.g. *F*, *t*, *r*) with confidence intervals, effect sizes, degrees of freedom and *P* value noted<br>*Give P values as exact values whenever suitable.*
☒	☐	For Bayesian analysis, information on the choice of priors and Markov chain Monte Carlo settings
☐	☒	For hierarchical and complex designs, identification of the appropriate level for tests and full reporting of outcomes
☒	☐	Estimates of effect sizes (e.g. Cohen's *d*, Pearson's *r*), indicating how they were calculated

Our web collection on statistics for biologists contains articles on many of the points above.

Software and code

Policy information about availability of computer code

Data collection	For phylogenetic inference, host mitochondrial genomes were extracted from metagenomic assemblies and aligned using MUSCLE (v3.8.1551). To infer the relationships between the six corresponding Stammera capleta symbionts, 61 single-copy core genes identified by anvi'o (v8.1-dev) and present in all symbionts were extracted from each Stammera capleta genome and aligned using MUSCLE (v3.8.1551).<br><br>For RNA sequencing data, adapter removal and quality filtering of raw reads were performed in Trimmomatic (v0.36) and FastQC (v0.12.0). Filtered reads were mapped to the Chelymorpha alternans genome using HISAT2 (v2.2.0). Gene counts were obtained using htseq-count (v0.11.5).
Data analysis	For phylogeny analyses, a concatenated alignment of 15 host mitochondrial genes was partitioned to assign the most appropriate substitution model to each gene using PartitionFinder2 (v2.1.1.). Phylogenetic analyses were performed using Maximum Likelihood (ML) in RAxML-NG (v1.2.0). To infer the relationships between the six corresponding symbionts, a concatenated alignment of 61 single-copy core genes was included to construct an ML phylogeny using RAxML-NG (v1.2.0). The best-fit substitution model was selected using PartitionFinder2 (v2.1.1.). The tree reconciliation software eMPRess GUI (v1.0.) was applied to investigate the evolutionary relationship between the six beetle species and their symbionts.<br><br>For symbiont genetic distance analyses, a dual approach was used to evaluate sequence similarity and structural gene organization across symbiont genomes. The Average Nucleotide Identity (ANI) between Stammera capleta genomes was calculated using PyANI (v2.4.0), as implemented in anvi'o (v8.1-dev), to assess symbiont relatedness. Gene collinearity across different Stammera capleta lineages was analyzed with MCScanX (v1.0) and SynTracker (v1.1.3). Collinearity scores were then calculated using the collinearity scripts from Nowell and colleagues (doi:10.5281/ZENODO.891288). SynTracker was used to calculate the Average Pairwise Synteny score (APSS).

For RNAseq analyses, read counts were normalized by the DESeq2's median of ratios established in the DESeq2 package. The likelihood ratio test (LRT), implemented in DESeq2, was used to test for differences in host gene expression across treatments. Host genes were considered significantly differentially expressed at adjusted p-value (p adj) < 0.05 and a fold-change > 2. Volcano plots were generated using the ggplot2 and dplyr packages for visualizing transcriptome data. We compared the differentially expressed gene (DEG) profile between treatments by testing for significant clusters using a permuted multivariate analysis of variance (PERMANOVA) and applying the function vegan::adonis(). The batch effect was removed using the LIMMA package. DEGs were further annotated for immune functions using the C. alternans immune gene reference set, which was derived from the 4IN database (Innate Immunity Genes in Insects: http://bf2i300.insa-lyon.fr:443/home). A heatmap of the log2(x+1) normalized counts was then constructed to illustrate host DEG expression patterns in response to colonization by S. capleta symbionts. Analyses were performed in R (v. 3.5.3).

To assess the colonization efficiency of different S. capleta species in C. alternans as a novel host, Fisher's Exact test was used to assess the effect of treatment (untreated, aposymbiotic, re-infected, or cross-infected) on symbiont infection frequency in larval foregut symbiotic organs. Pairwise comparisons between treatments were performed using the pairwise.prop.test() function with Holm correction to adjust for multiple testing. Abundances of different S. capleta symbionts in the foregut symbiotic organs of 5-day-old C. alternans larvae were analyzed using a generalized linear model with a quasi-Poisson error structure and a log-link function. The treatment, replicate, and experimental block were included in the model as fixed factors. Tukey's HSD pairwise comparisons were performed using the glht() function with Bonferroni corrections. The survival of C. alternans beetles into adulthood was analyzed with a Cox mixed-effect model testing for the effect of experimental treatments (untreated, aposymbiotic, re-infected or cross-infected) and replicates with experimental blocks as a random effect, and using the coxme package. A pairwise comparison between treatments was performed using the pairwise_survdiff() function with Bonferroni corrections in the survminer package. The survival data was visualized by computing the Kaplan–Meier survival functions. Spearman's rank correlations were used to test the relationship between larval survivorship and symbiont genetic distance (Average Nucleotide Identity or collinearity score) after testing the data distribution.

To compare the expression level of differentially expressed genes after colonization of foregut symbiotic organs by S. capleta symbionts, normalized transcripts were analyzed using either a negative binomial generalized linear model implemented by the glm.nb() function, or a general linear model framework depending on the data distribution and after verification that model assumptions were respected. In these statistical models, treatment and replicate were considered as fixed factors. Tukey's HSD pairwise comparisons were performed using the glht () function with Bonferroni corrections. Abundances of different S. capleta symbionts in the foregut symbiotic organs of 5-day-old C. alternans larvae (RNA-seq experiment) were analyzed using a general linear model after appropriate data transformation and verification of model assumptions. Treatment, replicate, and their interaction were included as fixed factors, and pairwise comparisons were conducted using Tukey's HSD with Bonferroni corrections via the glht() function. Fisher's Exact test was used to assess the effect of treatment (re-infected or cross-infected) on the proportion of S. capleta reads mapped to the symbiont genome relative to total reads mapped. Pairwise comparisons between treatments were performed using the pairwise.prop.test() function with Holm correction to adjust for multiple testing. Akaike information criterion was used for model selection.

The effect of experimental treatments (re-infected or cross-infected) on symbiont infection frequencies in foregut symbiotic organs or ovary-associated glands of C. alternans females 5- and 15-days post-emergence was analyzed using generalized linear models with a binomial error structure and a logit-link function. In these statistical models, treatment, time, and their interaction were considered as fixed factors. The effect of experimental treatments (re-infected or cross-infected adults) on symbiont infection frequency in their offspring was analyzed using a generalized linear model with a binomial error structure and a logit-link function, and the treatment was considered a fixed factor.

To compare the population dynamics of native and non-native bacteria in the foregut symbiotic organs of dual-infected beetles during development, their abundance was analyzed using a general linear model framework after verification that model assumptions were respected. The time, symbiont, replicate, experimental block, and the interaction between the time and symbiont were used as fixed factors. A pairwise comparison of the interaction was performed using least-squares means with Tukey corrections. Given that the data associated with adult foregut symbiotic organs include both females and males, the effect of sex on S. capleta population dynamics was determined by fitting a negative binomial generalized linear model implemented by the glm.nb() function. Beetle sex and symbiont were considered as fixed factors. The effect of beetle development time on the abundance of native and non-native symbionts was analyzed using general linear models after checking that model assumptions were respected and using time as a fixed factor. Tukey's HSD pairwise comparisons were performed using the glht() function with Bonferroni corrections. The impact of dual-infected beetles on S. capleta infection frequencies in their offspring was analyzed using a generalized linear model with a binomial error structure and a logit-link function, and the treatment was considered a fixed factor. Akaike information criterion was used to select all previous models.

Statistical analyses were performed in R (v. 3.5.3) using the prop.test() function to obtain the 95% confidence intervals for a binomial distribution, the survival package for survival analyses, multcomp and lsmeans packages for pairwise comparisons, MASS package for negative binomial generalized linear models, and ggplot2 for producing figures.

The R scripts generated in this study have been deposited in figshare [https://doi.org/10.6084/m9.figshare.28622969].

For manuscripts utilizing custom algorithms or software that are central to the research but not yet described in published literature, software must be made available to editors and reviewers. We strongly encourage code deposition in a community repository (e.g. GitHub). See the Nature Portfolio guidelines for submitting code & software for further information.

Data

Policy information about availability of data

All manuscripts must include a data availability statement. This statement should provide the following information, where applicable:

- Accession codes, unique identifiers, or web links for publicly available datasets
- A description of any restrictions on data availability
- For clinical datasets or third party data, please ensure that the statement adheres to our policy

Data Availability:
The raw reads of the RNA sequencing data generated in this study have been deposited in the National Center for Biotechnology Information (NCBI) SRA under accession codes SRR31763289-SRR31763297 [https://www.ncbi.nlm.nih.gov/bioproject/?term=PRJNA1199456]. The raw data generated in this study have been deposited in figshare [https://doi.org/10.6084/m9.figshare.28622969]. Source data are provided with this paper.

Code Availability:
The R scripts generated in this study have been deposited in figshare [https://doi.org/10.6084/m9.figshare.28622969].

Research involving human participants, their data, or biological material

Policy information about studies with human participants or human data. See also policy information about sex, gender (identity/presentation), and sexual orientation and race, ethnicity and racism.

Reporting on sex and gender	n/a
Reporting on race, ethnicity, or other socially relevant groupings	n/a
Population characteristics	n/a
Recruitment	n/a
Ethics oversight	n/a

Note that full information on the approval of the study protocol must also be provided in the manuscript.

Field-specific reporting

Please select the one below that is the best fit for your research. If you are not sure, read the appropriate sections before making your selection.

☐ Life sciences ☐ Behavioural & social sciences ☒ Ecological, evolutionary & environmental sciences

For a reference copy of the document with all sections, see nature.com/documents/nr-reporting-summary-flat.pdf

Life sciences study design

All studies must disclose on these points even when the disclosure is negative.

Sample size	n/a
Data exclusions	n/a
Replication	n/a
Randomization	n/a
Blinding	n/a

Behavioural & social sciences study design

All studies must disclose on these points even when the disclosure is negative.

Study description	n/a
Research sample	n/a
Sampling strategy	n/a
Data collection	n/a
Timing	n/a
Data exclusions	n/a
Non-participation	n/a
Randomization	n/a

Ecological, evolutionary & environmental sciences study design

All studies must disclose on these points even when the disclosure is negative.

Study description	We investigated the specificity of a co-diversified symbiosis by examining the streamlined partnership between tortoise leaf beetles (Chrysomelidae: Cassidinae) and their digestive bacterial symbiont, Candidatus Stammera capleta.
Research sample	The tortoise beetle-Stammera capleta partnership offers a tractable experimental framework to test fidelity in an obligate symbiosis, given the microbe's unique transmission route and colonization dynamics. Despite its drastically reduced genome (~0.25 Mb) and restricted localization within foregut symbiotic organs, Stammera capleta is vertically transmitted through egg-associated spheres. Manipulating these spheres allowed us to experimentally exchange Stammera capleta between beetle species to demonstrate that heritable endosymbionts can be exchanged between host species, non-native symbionts can successfully colonize, and, to varying degrees, rescue survivorship in a novel host, but that, ultimately, a high level of fidelity governs this partnership.
Sampling strategy	Sample size was predetermined on the basis of published studies, pre-experiments, the difficulty of experimental manipulations, and in-house expertise.
Data collection	Beetle egg masses were separated into different experimental treatments depending on the experiment: untreated control, aposymbiotic, re-infected with the native symbiont, and cross-infected with symbionts from different beetle species.<br><br>The experiments were generally conducted with between 3 and 10 replicates, ensuring a high level of reliability and consistency in the results. Typically, several individuals were included in each replicate to minimize variation. The experiments were performed under controlled laboratory conditions to eliminate environmental factors and ensure that results were due to the experimental variables rather than external influences.<br><br>The data were mainly recorded by Dr. Pons, with some assistance from the co-authors.<br><br>Whole beetles or foregut symbiotic organs dissected from embryos, larvae, or adults were collected to perform various techniques, such as fluorescence in situ hybridization (FISH), molecular techniques (polymerase chain reaction (PCR), quantitative polymerase chain reaction (qPCR)), or genomic approaches (RNA sequencing).<br><br>For the fitness experiment, insects were monitored daily to assess the impact of non-native symbionts on larval survivorship.<br><br>Published genomic data were used to perform phylogeny analyses and evaluate sequence similarity and structural gene organization across symbiont genomes.
Timing and spatial scale	The experiments began in 2021 and ended in 2025 at a regular interval. Each part of the study was carried out progressively to answer new questions raised.<br><br>In the case of the fitness experiments, insects were monitored daily.<br><br>For some experiments, several collection periods (experimental blocks) were performed to add more replicates to the different conditions or add experimental conditions to validate our results. Given the difficulty of generating the treatments (aposymbiotic eggs, eggs whose caplets were removed but re-infected with the native bacterium, or eggs whose caplets were removed but cross-infected with Stammera capleta-bearing spheres collected from different beetle species), not all replicates could be produced at the same time. These different experimental blocks were included in the statistical models where possible.
Data exclusions	No data were excluded from the analyses.
Reproducibility	N/A
Randomization	All samples and conditions were allocated randomly.
Blinding	Blinding was not relevant to the study, since all treatments were monitored equally.

Did the study involve field work? ☒ Yes ☐ No

Field work, collection and transport

Field conditions	Field work was conducted in several countries (temperate and tropical regions) to collect tortoise beetles (Chrysomelidae: Cassidinae).
Location	The beetle collection was carried out in Panama (latitude: 9.12, longitude: -79.70), France (latitude: -21.03, longitude: 55.22), and Germany (latitude: 48.52, longitude: 9.058).
Access & import/export	The beetles were collected using containers with air-permeable caps and transported with a leaf of their host plant to ensure their well-being during transport. This study complies with Nagoya Protocol regulations in the Republic of Panama and France, according to the following permits: ARG-027-2023 (issued by Autoridad Nacional del Ambiente (ANAM) / Autoridad Nacional de los Recursos Naturales (ANRN)) and TREL2302365S/693 (issued by Ministère de la Transition écologique et de la Cohésion des territoires /

	Direction générale de l'aménagement, du logement et de la nature / Direction de l'eau et de la biodiversité / Bureau de l'encadrement des impacts sur la biodiversité on 07/04/2023).
Disturbance	We ensured that these beetles were not endangered species by consulting local experts, and only a few specimens (1 to 3 individuals per species) were collected to minimize the impact on local populations. We also took care to follow best collection practices to avoid disturbing the natural habitat.

Reporting for specific materials, systems and methods

We require information from authors about some types of materials, experimental systems and methods used in many studies. Here, indicate whether each material, system or method listed is relevant to your study. If you are not sure if a list item applies to your research, read the appropriate section before selecting a response.

Materials & experimental systems

n/a	Involved in the study
☒	☐ Antibodies
☒	☐ Eukaryotic cell lines
☒	☐ Palaeontology and archaeology
☐	☒ Animals and other organisms
☒	☐ Clinical data
☒	☐ Dual use research of concern
☒	☐ Plants

Methods

n/a	Involved in the study
☒	☐ ChIP-seq
☒	☐ Flow cytometry
☒	☐ MRI-based neuroimaging

Antibodies

Antibodies used	n/a
Validation	n/a

Eukaryotic cell lines

Policy information about cell lines and Sex and Gender in Research

Cell line source(s)	n/a
Authentication	n/a
Mycoplasma contamination	n/a
Commonly misidentified lines (See ICLAC register)	n/a

Palaeontology and Archaeology

Specimen provenance	n/a
Specimen deposition	n/a
Dating methods	n/a

☐ Tick this box to confirm that the raw and calibrated dates are available in the paper or in Supplementary Information.

Ethics oversight	n/a

Note that full information on the approval of the study protocol must also be provided in the manuscript.

Animals and other research organisms

Policy information about studies involving animals; ARRIVE guidelines recommended for reporting animal research, and Sex and Gender in Research

Laboratory animals	Six tortoise beetles (Chrysomelidae: Cassidinae) were maintained in a greenhouse at the Max Planck Institute for Biology in Tübingen, Germany (Chelymorpha alternans, Chelymorpha bullata, Chelymorpha gressoria, Aspidimorpha quinquefasciata, Acromis sparsa, and Cassida rubiginosa). All stages (eggs, larvae, and adults) of these insects were used in this study.

Wild animals	The six tortoise beetles maintained in the laboratory were collected in Panama, France, and Germany using containers with air-permeable caps and transported with a leaf of their host plant. Only a few reproductive adults (1 to 3) were collected per species. The insects were then placed in mesh containers along with their host plants in a greenhouse and used in this study.
Reporting on sex	Sex can only be determined in the adult stage. Females are oval with a slight indentation at the edge of the pronotum and elytra, while males are more circular, with pronounced dentition between the pronotum and elytra. Both sexes are necessary for their maintenance in the laboratory. Sex was considered in experiments using adult insects.
Field-collected samples	The insects were reared in mesh containers with their host plants in a greenhouse. Experimental treatments were maintained in climate chambers at a constant temperature of 26°C, humidity of 60%, and long light regimes (14.30 h/9.30 h light/dark cycles).
Ethics oversight	No ethical approval or guidance was required, as insect experimentation is not governed by animal experimentation regulations.

Note that full information on the approval of the study protocol must also be provided in the manuscript.

Clinical data

Policy information about clinical studies

All manuscripts should comply with the ICMJE guidelines for publication of clinical research and a completed CONSORT checklist must be included with all submissions.

Clinical trial registration	n/a
Study protocol	n/a
Data collection	n/a
Outcomes	n/a

Dual use research of concern

Policy information about dual use research of concern

Hazards

Could the accidental, deliberate or reckless misuse of agents or technologies generated in the work, or the application of information presented in the manuscript, pose a threat to:

No	Yes	
☒	☐	Public health
☒	☐	National security
☒	☐	Crops and/or livestock
☒	☐	Ecosystems
☒	☐	Any other significant area

Experiments of concern

Does the work involve any of these experiments of concern:

No	Yes	
☒	☐	Demonstrate how to render a vaccine ineffective
☒	☐	Confer resistance to therapeutically useful antibiotics or antiviral agents
☒	☐	Enhance the virulence of a pathogen or render a nonpathogen virulent
☒	☐	Increase transmissibility of a pathogen
☒	☐	Alter the host range of a pathogen
☒	☐	Enable evasion of diagnostic/detection modalities
☒	☐	Enable the weaponization of a biological agent or toxin
☒	☐	Any other potentially harmful combination of experiments and agents

Plants

Seed stocks	n/a
Novel plant genotypes	n/a
Authentication	n/a

ChIP-seq

Data deposition

☐ Confirm that both raw and final processed data have been deposited in a public database such as GEO.

☐ Confirm that you have deposited or provided access to graph files (e.g. BED files) for the called peaks.

Data access links *May remain private before publication.*	n/a
Files in database submission	n/a
Genome browser session (e.g. UCSC)	n/a

Methodology

Replicates	n/a
Sequencing depth	n/a
Antibodies	n/a
Peak calling parameters	n/a
Data quality	n/a
Software	n/a

Flow Cytometry

Plots

Confirm that:

☐ The axis labels state the marker and fluorochrome used (e.g. CD4-FITC).

☐ The axis scales are clearly visible. Include numbers along axes only for bottom left plot of group (a 'group' is an analysis of identical markers).

☐ All plots are contour plots with outliers or pseudocolor plots.

☐ A numerical value for number of cells or percentage (with statistics) is provided.

Methodology

Sample preparation	n/a
Instrument	n/a
Software	n/a
Cell population abundance	n/a
Gating strategy	n/a

☐ Tick this box to confirm that a figure exemplifying the gating strategy is provided in the Supplementary Information.

Magnetic resonance imaging

Experimental design

Design type: n/a

Design specifications: n/a

Behavioral performance measures: n/a

Acquisition

Imaging type(s): n/a

Field strength: n/a

Sequence & imaging parameters: n/a

Area of acquisition: n/a

Diffusion MRI: ☐ Used ☒ Not used

Preprocessing

Preprocessing software: n/a

Normalization: n/a

Normalization template: n/a

Noise and artifact removal: n/a

Volume censoring: n/a

Statistical modeling & inference

Model type and settings: n/a

Effect(s) tested: n/a

Specify type of analysis: ☐ Whole brain ☐ ROI-based ☐ Both

Statistic type for inference (See Eklund et al. 2016): n/a

Correction: n/a

Models & analysis

n/a	Involved in the study
☒	☐ Functional and/or effective connectivity
☒	☐ Graph analysis
☒	☐ Multivariate modeling or predictive analysis

Functional and/or effective connectivity: n/a

Graph analysis: n/a

Multivariate modeling and predictive analysis: n/a
